# Charged Small Molecule Binding to Membranes in MD Simulations Evaluated against NMR Experiments

**DOI:** 10.1021/acs.jpcb.2c05024

**Published:** 2022-09-05

**Authors:** Ricky Nencini, O. H. Samuli Ollila

**Affiliations:** Institute of Biotechnology, University of Helsinki, 00014 Helsinki, Finland

## Abstract

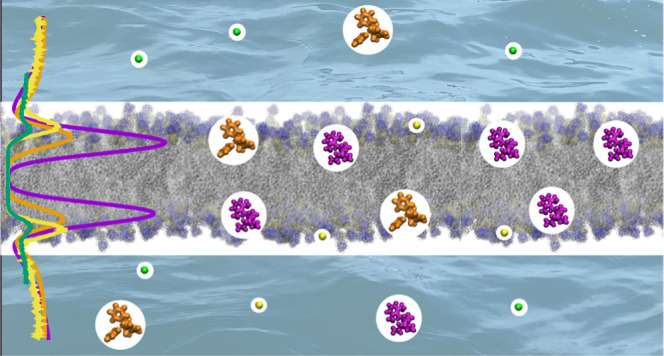

Interactions of charged molecules with biomembranes regulate many of their biological activities, but their binding affinities to lipid bilayers are difficult to measure experimentally and model theoretically. Classical molecular dynamics (MD) simulations have the potential to capture the complex interactions determining how charged biomolecules interact with membranes, but systematic overbinding of sodium and calcium cations in standard MD simulations raises the question of how accurately force fields capture the interactions between lipid membranes and charged biomolecules. Here, we evaluate the binding of positively charged small molecules, etidocaine, and tetraphenylphosphonium to a phosphatidylcholine (POPC) lipid bilayer using the changes in lipid head-group order parameters. We observed that these molecules behave oppositely to calcium and sodium ions when binding to membranes: (i) their binding affinities are not overestimated by standard force field parameters, (ii) implicit inclusion of electronic polarizability increases their binding affinity, and (iii) they penetrate into the hydrophobic membrane core. Our results can be explained by distinct binding mechanisms of charged small molecules with hydrophobic moieties and monoatomic ions. The binding of the former is driven by hydrophobic effects, while the latter has direct electrostatic interactions with lipids. In addition to elucidating how different kinds of charged biomolecules bind to membranes, we deliver tools for further development of MD simulation parameters and methodology.

## Introduction

Binding affinities of charged molecules, such as drugs, amino acids, ions, and pollutants, on cellular membranes, regulate many of their biological functions.^1−6^ For example, signaling domains in proteins often contain charged residues and interact with membranes in an ion-dependent manner,^1,4−6^ translocation of drugs through membranes depends on their charge state,^7^ and bioaccumulation of charged pollutants can be related to their membrane affinity.^3,8^ Such processes are particularly poorly understood for charged molecules because their binding to membranes is significantly more difficult to study than for neutral molecules. Therefore, better understanding and predictive models for membrane binding of charged molecules would benefit applications in a wide range of fields, such as designing drugs with better translocation properties, and understanding cell signaling and bioaccumulation of potentially toxic molecules.^3,6,7,9,10^

For neutral molecules, the water–oil (often octanol) partition coefficients correlate well with the binding data on model membranes and their binding affinities can be captured equally well by theoretical models with atomistic and continuum-level descriptions.^9,11−13^ However, measurements of water–oil partition coefficients are problematic for charged molecules due to the charge neutralization in both phases.^14,15^ Furthermore, experimental data on lipid binding affinity is more scarce, and a complex electrostatic environment at membrane interfaces, created by dipoles of lipids and water molecules, complicates theoretical analyses.^14,15^ Incorporation of the membrane dipole potential in continuum models improves the results,^14^ but atomistic resolution description is needed to fully capture the complexity of electrostatic and other interactions between lipids, water, and charged molecules at membrane interfaces.

However, the correct description of interactions between charged water-soluble molecules and membranes has been challenging for atomistic resolution models employed in molecular dynamics (MD) simulations. Canonical force fields tend to overestimate the binding affinities of sodium and calcium to membranes,^16,17^ yet this can be improved by electronic continuum correction (ECC)^16,18−20^ or additional repulsive potential (NBFIX).^17,21,22^ Also lipid–protein interactions depend on force field parameters,^23−26^ particularly for charged residues^27,28^ and their interactions with lipid head groups.^29,30^ Furthermore, charged residues in strongly membrane-bound peptides appear as potential sources of discrepancies in comparisons with NMR data^23,31^ and often lead to the largest deviations from experimental hydrophobicity scales.^27,28,32^ These findings raise the question of whether the interactions between charged molecules and membranes can be correctly captured in MD simulations without including electronic polarizability or other corrections. Therefore, it is currently not clear how accurately atomistic MD simulations can predict interactions between membranes and charged molecules, such as drugs, amino acids, or pollutants.

Sodium and calcium ion binding affinities to membranes with various compositions have been quantitatively evaluated in simulations using the changes in lipid head-group C–H bond order parameters,^16,20,33^ which depend on the accumulation of charges on the membrane.^34^ Here, we apply the same approach to quantify the binding affinities of etidocaine and tetraphenylphosphonium (TPP) ions to a phosphatidylcholine (POPC) lipid bilayer, serving as a model cellular membrane. Etidocaine is a clinically used local anesthetic and serves here as a model for charged drug binding to a membrane. TPP is a hydrophobic ion historically used to establish the concept of membrane dipole potential^35^ and serves as a model for charged aromatic compounds which are common among potentially bioaccumulating ions.^3^ Our results compare the relative binding affinities of these molecules to membranes with respect to sodium and calcium and test the quality of MD simulations regarding these affinities. Furthermore, we also investigate the binding mechanisms of these small molecules to membranes and the effect of implicit inclusion of electronic polarizability using ECC.

## Methods

### Force Field Parameters

For simulations with the standard force fields, CHARMM36 parameters^36^ from CHARMM-GUI^37−39^ were used for lipids. Parameters for etidocaine were generated with two standard approaches: SwissParam^40^ from https://www.swissparam.ch/, denoted here as CHARMM36-SwissParam, and Cgenff (CHARMM general force field)^41−43^ from https://cgenff.umaryland.edu/, denoted here as CHARMM36-ParamChem. For TPP, the standard approaches cannot be used since there are no parameters for the phosphonium atom. Therefore, we used CHARMM atom types together with the charges calculated using Gaussian using the density functional theory B3LYP functional and CHELPG scheme (charges from electrostatic potentials using a grid-based method),^44^ denoted here as CHARMM36-Qmcharges. Partial charges from our Gaussian calculation slightly deviated from the ones reported in the literature,^45^ see Figure S1 in the Supporting Information. Binding affinities to membranes were tested with both charges, but the results were similar, and only the results with our parameters from Gaussian (CHARMM36-Qmcharges) are shown. In another approach, denoted here as CHARMM36-ProteinFF, charges for TPP were obtained from the phenyl ring of phenylalanine in the CHARMM36 protein force field. All the systems were run with the TIP3P CHARMM36 water model.^46^

For simulations with ECC, we used our recently parameterized model compatible with CHARMM36 for POPC (PN-model in ref (47)). Following the simplest ECC approach,^48,49^ all partial charges in CHARMM36-ParamChem parameters of etidocaine (Table S1 in the Supporting Information) and CHARMM36-Qmcharges of TPP (Figure S1 in the Supporting Information) were scaled by 0.75. Partial charges used in all etidocaine and TPP simulations are listed in Figure S1 and Table S1 in the Supporting Information.

### Simulation Details

Lipid bilayers, consisting of 200 POPC molecules, were generated in CHARMM-GUI.^37−39^ Structures of etidocaine and TPP were built in Chimera or downloaded from https://zinc.docking.org/. Lipid bilayers were then surrounded by the desired number of etidocaine or TPP, which were placed in a 3D grid with equidistant positions to fill the whole space of the selected box size. The systems were then solvated with water molecules and neutralized with chloride counterions using GROMACS tools. The simulations were started from two different kinds of starting structures. The small molecules were either placed in the water phase or a snapshot from an already existing simulation was used. The latter approach was used for systems where all molecules were bound to the membrane, as described below. The numbers of water molecules were tuned to produce the desired concentrations. Equilibration of the systems was monitored by analyzing the number of bound particles as a function of time. The simulation times of individual trajectories ranged between approximately 100 ns and 5 μs, see Tables S3–S13. For selected systems, we prepared also starting structures with all molecules bound in a membrane and confirmed that the number of bound molecules converged to the same value as from a starting configuration with all molecules in the water phase, see Figure S5. The exact number of molecules and other details are listed in Tables S3–S13.

Systems were run using GROMACS software (versions 2018.6, 2018.8, 2019.3, 2021.1, 2021.4, and 2021.5).^50,51^ The temperature was coupled to 298 K corresponding to temperatures in experiments using a Nosé–Hoover thermostat.^52,53^ Particle mesh Ewald (PME) was used to calculate electrostatic interactions at distances longer than 1.2 nm.^54,55^ Lennard-Jones interactions were cut off at 1.2 nm. The pressure of 1 bar was maintained using a semi-isotropic Parrinello–Rahman barostat.^56^

### Analysis of Simulations

C–H bond order parameters were calculated from equation *S*_CH_ = ⟨3 cos θ – 1⟩/2, where θ is the angle between the C–H bond vector and the average is taken over the ensemble. Codes available in the NMRlipids project were used for order parameter calculations.^33^ To estimate the number of bound molecules to the bilayer, we first calculated how strongly the number of bound molecules to the membrane depends on the selected criteria for the distance from lipids and fraction of bound atoms. Then, the values at the point of weakest dependence (minimum of the gradient) were selected; see the Supporting Information for details. The density distributions along the membrane normal were calculated using a histogram method. Atom coordinates were centered around the center of mass of the POPC lipid molecules for every time frame, and a histogram of these centered positions was calculated with the bin width of 1/3 Å. The script utilizing the MDAnalysis module^57,58^ in Python is available at https://github.com/nencini/charged_molecules_binding. The potential of mean force (PMF) profiles were calculated from the density profiles ρ(*z*) using the inverse Boltzmann formula PMF = −*k*_b_*T* ln ρ(*z*). When calculating the number of contacts to specific regions (such as the phosphate group), two atoms were considered to be in contact when the distance between them was smaller than 0.325 nm. Permeation of particles through the membrane bilayer was analyzed by the program from Camilo et al.^59^ Instead of water oxygen used in the original work, the N14 atom was used for the analysis of permeation of etidocaine and the P atom was used for TPP molecules.

## Results and Discussion

### Evaluating the Charged Small Molecule Binding to Membranes in MD Simulations Using Lipid Head-Group Order Parameters

**Figure 1:**
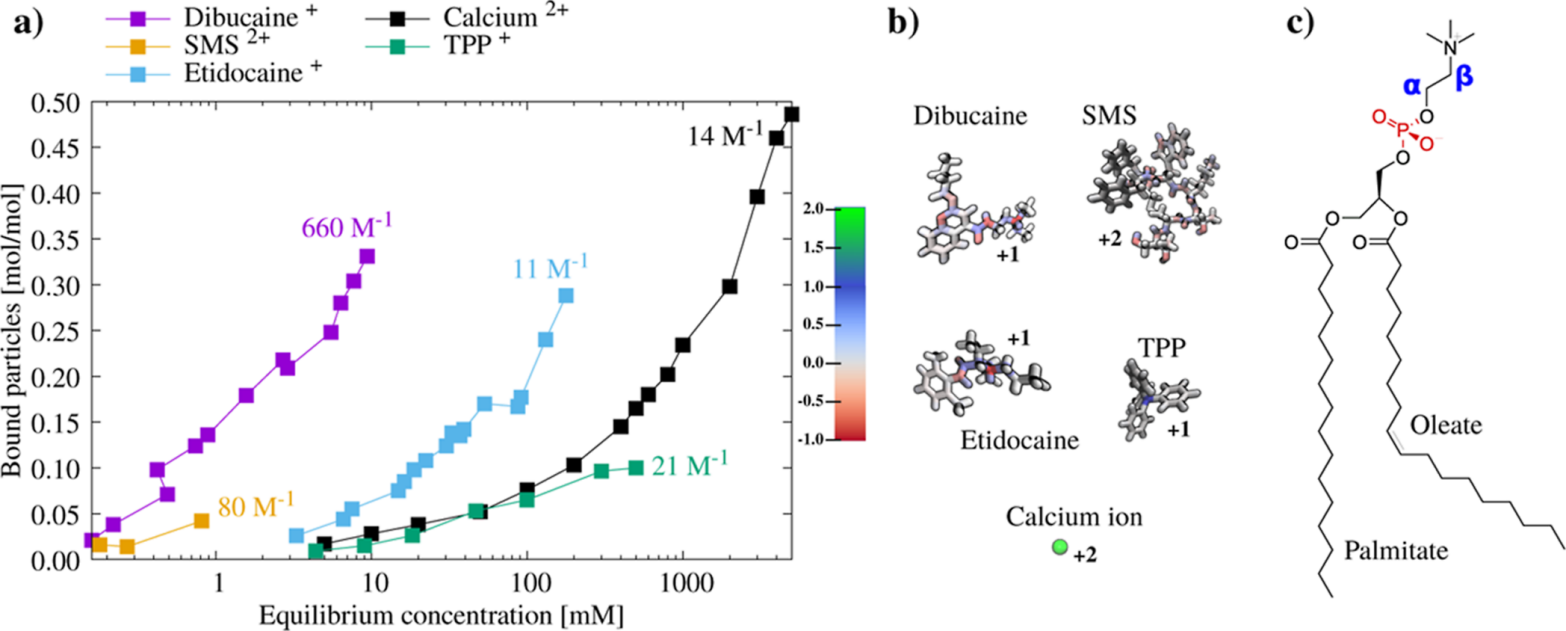
(a) Number of bound molecules per POPC lipid as a function of equilibrium concentration in the supernatant and binding coefficients for different charged molecules from the literature. The number of bound particles is determined by the difference in supernatant concentration before and after the addition of lipids. Concentrations are determined by UV spectroscopy (SMS,^62^ TPP,^63^ etidocaine,^64^ and dibucaine^64^) or atomic absorption spectroscopy (calcium ions^65^). The models used to determine binding coefficients and further details are shown in Table S2 in the Supporting Information. (b) Structures and charge distributions of the small molecules. For the chemical structure of etidocaine and TPP, see Figures S1 and S2 in the Supporting Information. (c) Structure of the POPC lipid molecule with the head-group α and β carbons labeled with blue and phosphate oxygens prone to calcium binding labeled with red.

Partition coefficients have been typically used to compare binding affinities of small molecules between MD simulations and experiments.^12,13,60^ Such values are available also for some charged small molecules^15^ but not for simple ions such as sodium or calcium. On the other hand, the affinities of ions to membranes have been reported using binding constants based on models assuming a certain binding stoichiometry for lipid–ion interactions, but these values strongly depend on the model used to interpret the experimental data.^61^ For example, the reported binding constants of calcium to neutral POPC membranes vary between 7 and 441 M^–1^.^61^ Furthermore, such binding constants may not even reflect the relative binding affinities of different molecules to membranes. This is exemplified in Figure 1 where the number of bound TPP (reported binding constant of 21 M^–1^) and calcium (reported binding constant of 14 M^–1^) ions per lipid molecule are similar, while the number of bound etidocaine molecules (reported binding constant of 11 M^–1^) is larger at similar bulk concentrations.

The unambiguity in reported binding constants can be circumvented by directly comparing how lipid head-group C–H bond order parameters change upon the addition of ions between simulations and experiments.^16^ These order parameters are proportional to the amount of charged molecules bound to the bilayer because binding of positive charges decreases their values (due to the orientation of the lipid head-group dipole more parallel to the membrane normal) and vice versa for negative charges.^16,34^ As we are interested in the relative binding affinities between charged small molecules and biologically relevant simple ions, such as calcium and sodium, we use this approach here. From the available experimental data for head-group order parameter changes upon binding of charged molecules,^66^ we chose to evaluate the binding affinities of etidocaine, TPP, dibucaine, and cyclic somatostatin (SMS) binding to a POPC lipid bilayer in simulations.

The approach has been successfully applied for sodium and calcium binding to various membranes,^16,18−20,33^ but care must be taken to confirm that the simulation setup is sufficiently close to experiments, particularly in terms of molecular concentrations in the system and equilibration of the binding. While changing the hydration level with constant ion concentration did not affect the conclusions in previous sodium and calcium simulations,^18^ the situation is more complex for small molecules with stronger binding affinities. The concentration of these molecules substantially decreases in bulk water upon binding to the membrane. In simulations with the hydration levels lower than in experiments, all solute molecules may bind to the membrane, thereby leading to the artificially underestimated bulk concentration. An ideal solution would be to use the same hydration level as in experiments, but this often leads to large boxes with substantial computational costs. Therefore, we estimated the minimal level of hydration, after which the results are not affected by the further addition of water while keeping the ion concentration in water constant.

To quantify the effect of hydration on the binding, we simulated systems with a fixed etidocaine concentration in simulation boxes with the z-dimension ranging from 5 to 81 nm. The lower limit corresponds to a typical size for membrane simulations, and the upper limit corresponds to the water–lipid ratio used in the experimental setup for etidocaine.^64^ For parameters predicting the weakest binding affinity, CHARMM36-SwissParam, the results are similar with box sizes above approximately 12 nm in the z-direction (Figures S4 and S5 in the Supporting Information). However, in simulations with CHARMM36-ParamChem and CHARMM36-ParamChem-ECC predicting stronger binding affinities, the dependence on the box size is observed also above z-dimensions of 12 nm. Further simulations with these parameters were, therefore, run using the large box with the *z*-dimension of approximately 81 nm, while boxes with the z-dimension of approximately 12 nm were used for other systems with weaker binding affinities to reduce the computational cost. Because binding of charged molecules to zwitterionic PC membranes substantially increases the lamellar repeat distance in a lipid bilayer stack,^67^ we consider such a large interbilayer space reasonable for the studies of strongly membrane-bound charged molecules.

The combination of the required large simulation boxes with the slow equilibration times makes simulations unfeasible for some strongly bound molecules. For example, bulk concentrations of the SMS peptide are in the sub-millimolar range in experiments^62^ which would require approximately 200,000 water molecules per peptide in simulations. On the other hand, binding or unbinding events were not observed for dibucaine during the 1360 ns simulation. Nevertheless, for etidocaine and TPP, the binding dynamic was sufficiently fast to enable to equilibrate the simulations within feasible time (1–5 μs) and simulation box z-dimension (12–80 nm) scales (Figures S4 and S5 in the Supporting Information). Therefore, we proceeded to evaluate the binding affinities of these molecules in MD simulations to membranes against the available lipid head-group order parameter data.

### Comparison of Etidocaine and TPP Binding to POPC Membranes between Simulations and Experiments

**Figure 2:**
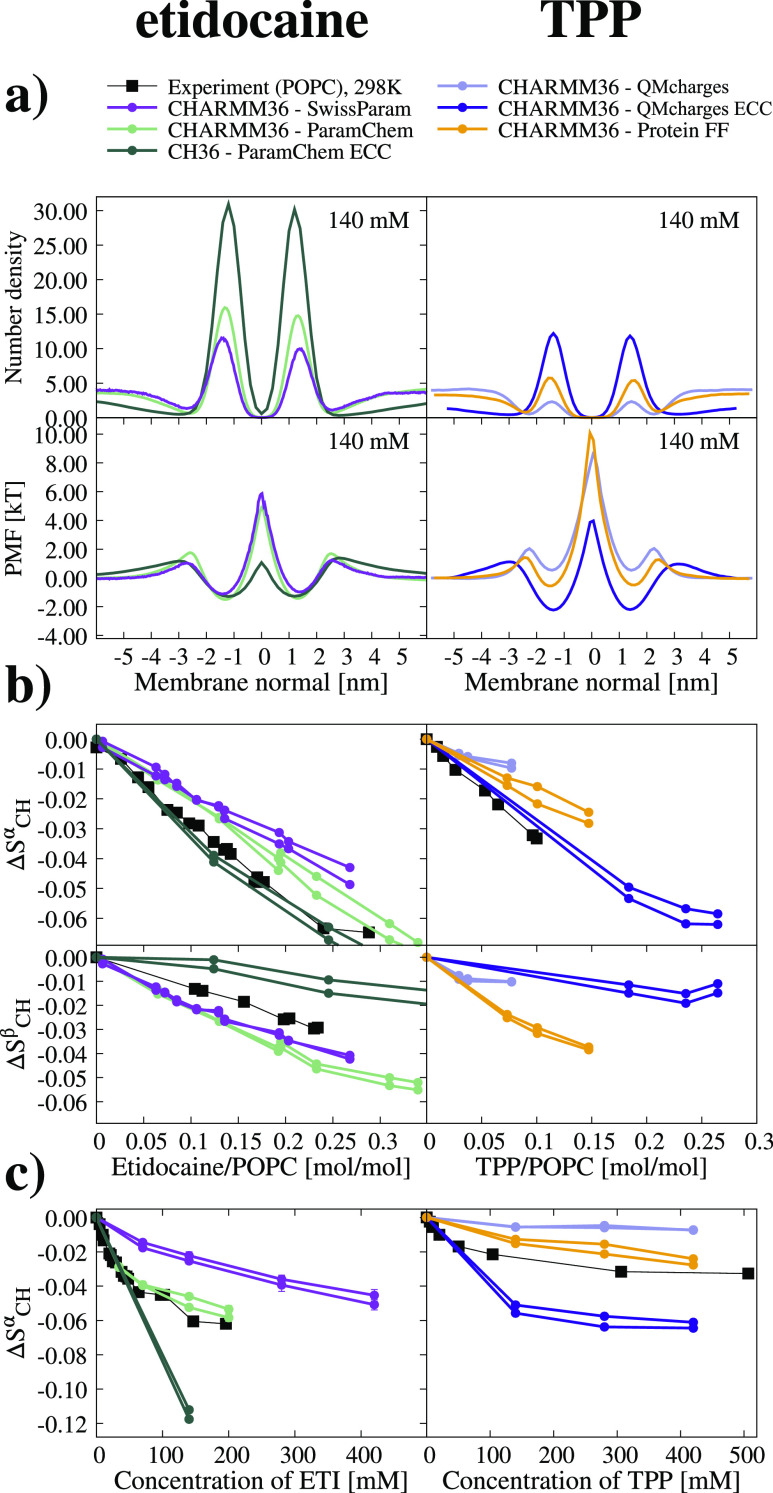
(a) Number densities and PMF profiles of etidocaine and TPP along the membrane normal from simulations with the small molecule concentration of 140 mM at 298 K. To emphasize the membrane region, the *x*-axis is limited between −6 and +6 nm, for the full density profile of etidocaine, see Figure S8 in the Supporting Information. Etidocaine results are on the left and TPP on the right throughout the figure. (b) Changes in the α and the β order parameters as a function of the bound etidocaine and TPP per lipid in the POPC membrane. (c) Changes in the POPC head-group α-carbon order parameters as a function of small molecule concentration with respect to water from simulations and experiments for etidocaine^64^ and TPP.^63^ Error bars from simulations are not visible for most order parameters as they are smaller than the point size.

The binding behavior of etidocaine and TPP molecules to POPC membranes predicted by different force field parameters is illustrated in Figure 2a using the density profiles along the membrane normal and PMF curves derived from therein. For etidocaine, CHARMM36-SwissParam predicts the weakest binding affinity, followed by the CHARMM36-ParamChem and CHARMM36-Paramchem ECC. The weakest affinity for TPP ions is predicted by CHARMM36-QMcharges, followed by CHARMM36-ProteinFF and CHARMM36-QMcharges ECC.

To evaluate the simulation predictions against experiments, we calculated the C–H bond order parameters of α and β segments in the POPC head group, which are proportional to the amount of bound charge in a membrane.^34^ As shown in Figure 2b, a linear decrease in these order parameters is observed as a function of the number of bound etidocaine or TPP molecules to a POPC membrane. This trend is observed in both MD simulations and NMR experiments,^63,66^ but the slope of the decrease depends on force field parameters used in a simulation and deviates from experiments in all cases except for the α carbon in ECC simulations. This indicates that the interactions of these molecules with a POPC lipid membrane are not accurately captured by any of these simulations.

Nevertheless, we consider that the responses of the α-carbon order parameters are sufficiently close to experiments to be used for the validation of etidocaine and TPP binding affinities in simulations, yet the observed inaccuracy should be taken into account when interpreting the results. The decrease in the α-carbon order parameter as a function of etidocaine concentration in water in Figure 2c correlates with the binding affinity in Figure 2a, being weakest for the CHARMM36-SwissParam, followed by the CHARMM36-ParamChem and CHARMM36-ParamChem ECC. Comparison with the experimental data suggests that CHARMM36-ParamChem simulations predict the etidocaine binding affinity closest to experiments, while CHARMM36-SwissParam predicts too weak and CHARMM36-ParamChem ECC too strong binding. A similar comparison for TPP suggests that CHARMM36-ProteinFF prediction is closest to experiments, while CHARMM36-QMcharges predicts too weak binding, which is then overestimated after applying the ECC. However, because QM-derived partial charges are presumably more realistic, the better results with CHARMM36-ProteinFF parameters probably originate from the cancellation of errors.

In conclusion, none of the parameters generated with the standard approaches for etidocaine or TPP predicted the overbinding of these molecules to POPC membranes, while some parameters predicted too weak binding affinity. Therefore, our results suggest that the previously observed overbinding of sodium and calcium ions to membranes in canonical MD simulation force fields^16^ is not a general feature for all positively charged biomolecules.

### Effect of Electronic Polarizability and Binding Mechanism of Etidocaine and TPP to Membranes

The most likely source for the discrepancies in MD simulations of charged small-molecule binding on membranes is the missing electronic polarizability. While polarizable force fields are available, such as CHARMM36-Drude,^68^ they cannot yet capture the lipid head-group conformational ensembles with sufficient accuracy to enable the usage of head-group order parameters for the evaluation of small-molecule binding affinities.^69^ On the other hand, the implicit inclusion of electronic polarizability by scaling the partial charges of atoms in an approach known as ECC^48^ has been shown to correct the overestimated calcium binding to membranes containing charged and zwitterionic lipids.^18−20^ Therefore, we decided to study the effect of electronic polarizability by applying the ECC to CHARMM36-ParamChem parameters of etidocaine and to CHARMM36-QMcharges of TPP. These were used with the recently introduced parameters for POPC where ECC was applied to the CHARMM36 lipid force field.^47^

Applying ECC increases the binding affinities of both the etidocaine and the TPP molecules to POPC bilayers, leading to an intensified decrease in order parameters in Figure 2a. On the other hand, ECC makes the β-carbon order parameter less sensitive, and α-carbon slightly more sensitive, to both etidocaine and TPP. This brings the responses to the number of bound molecules in Figure 2b closer to experiments, although the decrease in β-carbon upon the addition of etidocaine is now slightly underestimated. In conclusion, ECC potentially improves the interactions of etidocaine and TPP with the POPC head group. However, it introduces too strong binding affinity, emphasizing the need for further optimization of force field parameters to accurately capture the binding details of TPP, etidocaine, and other charged small molecules to lipid membranes.

Notably, the effect of ECC on the binding affinities of etidocaine and TPP is opposite to its effect on the calcium-binding affinity which decreased upon applying ECC.^18−20^ This can be explained by the different binding mechanisms of calcium and small molecules, such as etidocaine and TPP. The main driving forces for calcium binding to a membrane with PC lipids are the direct electrostatic interactions with phosphate oxygens,^18^ as also seen in Table 1, where the majority of calcium–lipid interactions occur with phosphate oxygens. For small molecules with charges surrounded by hydrophobic moieties, such as etidocaine and TPP, the hydrophobic effect is the most likely driving force for their membrane binding. In the case of calcium, the inclusion of ECC reduces the electrostatic attraction with phosphate oxygen, which is the most likely reason for the reduced binding affinity. Indeed, the reduction in interactions with phosphate oxygen due to ECC is observed for calcium in Table 1 but not for etidocaine or TPP. On the other hand, the scaling of total charge by ECC makes etidocaine and TPP less soluble in water, thereby increasing their binding affinity to membranes. In conclusion, the binding behavior of monoatomic ions, such as calcium, to membranes differs from small molecules, such as etidocaine and TPP, due to their different binding mechanisms.

**Table 1:** Number of Contacts with Any Lipid Atom per Bound Molecule and Percentage of the Contacts with Phosphate Oxygens^a^

molecule	contacts/bound molecule	P-contacts/all contacts [%]
Ca^2+^ CHARMM36 no NBFIX	3.9	90.6
Ca^2+^ CHARMM36 ECC	2.2	64.6
Ca^2+^ Lipid14	7.6	67.3
Ca^2+^ Lipid14 ECC	3.5	64.9
ETI CHARMM36-ParamChem	17.4	10.0
ETI CHARMM36-ParamChem ECC	18.5	11.1
TPP CHARMM36-QMcharges	17.0	7.9
TPP CHARMM36-QMcharges ECC	18.74	11.2

a CHARMM36-based simulations with 450 mM CaCl_2_^47^ available at refs (70) and (71) and Amber-based Lipid14 simulations^18^ with 467 mM CaCl_2_ available at refs (72) and (73) were used. Results from simulations with 70 mM Etidocaine and 140 mM TPP are shown.

### Comparison of Sodium, Calcium, Etidocaine, and TPP Binding to Membranes

Experimental methods can provide accurate information on binding affinities of charged molecules to membranes, but it is not straightforward to define a concentration and model-independent binding coefficient that would correctly describe the binding affinity of charged biomolecules to membranes with a single number, as demonstrated in Figure 1. While the relative binding affinities can be judged by examining the whole experimental binding isotherm, detailed information such as the depth of binding or the potential mechanism of penetration remains inaccessible from the experimental data alone. On the other hand, simulations that reproduce the experimental data can be used to interpret such properties from the experimental data.

**Figure 3:**
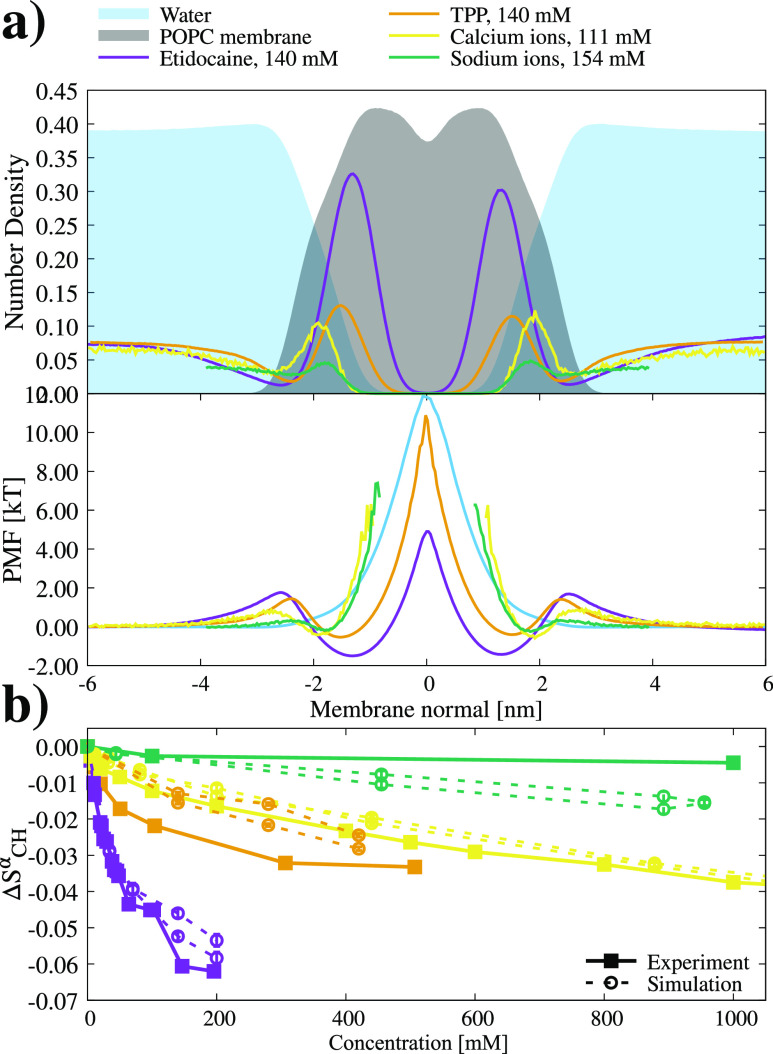
(a) Molecule number density profiles and PMF profile from simulations giving the most realistic binding affinities of etidocaine (CHARMM36-ParamChem), TPP (CHARMM36-ProteinFF), calcium, and sodium (simulations with ECC correction^47^) according to the head-group order parameters responses. Results from CHARMM36 with ECC applied^47^ are shown for calcium and sodium,^70,74^ but simulation with ECC applied to Amber parameters would give essentially the same conclusions.^18^ (b) Experimental NMR α-carbon order parameter responses to etidocaine, TPP, calcium, and sodium ions are shown as full lines together with selected most realistic simulations shown as dashed lines.

Because a single force field that would correctly reproduce the binding of all charged molecules to membranes is not yet available, we selected the best available models from this and our previous study^47^ to compare the binding of sodium, calcium, etidocaine, and TPP to a POPC membrane. The density profiles, PMFs, and α-carbon order parameter decrease compared with experiments for these simulations are shown in Figure 3. In these simulations, the sodium exhibits weaker binding affinity than other molecules as expected for simple monovalent ions.^16^ Calcium and TPP bind with similar affinities, while etidocaine exhibits the strongest binding. These relative binding affinities are in line with the experimentally determined number of bound particles in Figure 1 and measured order parameters but not with the reported binding coefficients. Despite the similar binding affinities, TPP penetrates deeper in the membrane than calcium. This can be explained by the different binding mechanisms of these molecules. Calcium ions bind to phosphate oxygens in the POPC head group, whereas the binding of TPP is driven by the hydrophobic effect. Similarly to TPP, also etidocaine penetrates deeper into a membrane. The PMF profiles in Figure 3 show a lower energy barrier at the membrane center for etidocaine and TPP than for water, sodium, and calcium. However, we did not observe any permeation events of etidocaine or TPP through the membrane. This is in contrast to water molecules, for which dozens of events were observed in each simulation. This can be explained by the larger size of etidocaine and TPP molecules increasing their probability to locate at the membrane center without permeating through the membrane. While we consider these observations as reasonable interpretations of the experimental data with the best available MD simulation models, we emphasize that the detailed interactions in these simulations may not be exactly correct since the β-carbon order parameter response to studied charged molecules was not in agreement with the experiment in Figure 2.

In conclusion, our results indicate that the binding affinities of charged drugs, etidocaine, dibucaine, and SMS, are significantly stronger to zwitterionic membranes than the binding affinities of sodium and calcium. Also, their binding mechanisms differ from that of monoatomic ions. Therefore, the binding of these drugs to membranes would be most likely not interfered by physiological ions. On the other hand, the binding of TPP could be interfered by calcium as their affinities are similar, yet their interrelated binding may be complicated as their binding mechanisms are different. MD simulations have the potential to model such complex behavior, but force field parameters correctly capturing both TPP and calcium-binding are not yet available. Furthermore, improvements in force fields would be needed to correctly model the binding of etidocaine to POPC head groups as the experimental response of β-carbon order parameters to the number of bound molecules is not captured by any simulation models in Figure 2b.

## Conclusions

While concentration and model-independent binding coefficients that would enable the comparison between the binding affinity of simple ions and charged small molecules to membranes are not available, we managed to evaluate the binding affinity of etidocaine and TPP to POPC lipid bilayers in MD simulations against experiments using the changes in lipid head-group-order parameters as done previously for sodium and calcium.^16^ However, the required size and length of simulations increase with increasing binding affinity, thereby setting practical limitations for the molecules that can be studied with this approach. For etidocaine and TPP, the simulation box sizes of 12–81 nm in membrane normal direction and time scales of 1–5 μs were sufficient, but the evaluation of SMS or dibucaine binding with higher affinities was not feasible within this work.

According to the evaluation based on lipid head-group order parameter changes, etidocaine and TPP binding affinities to a POPC membrane were slightly underestimated or close to experiments in simulations with standard CHARMM36-based force field parameters. This is in contrast with previous studies for sodium and calcium ions, where binding affinities were typically overestimated by canonical force fields.^16^ Therefore, our results suggest that force fields do not generally overestimate the binding of all positively charged molecules to membranes. Furthermore, the implicit inclusion of electronic polarizability using ECC increased the binding affinities of etidocaine and TPP to a POPC membrane, whereas calcium is known to behave oppositely.^18−20^ These observations can be explained by the different binding mechanisms of calcium and the small molecules with hydrophobic moieties. Calcium binds to the lipid phosphate oxygens via direct electrostatic interactions. The binding of small molecules is, on the other hand, driven by hydrophobic interactions. While ECC reduces the direct attraction between lipids and ions, it also reduces the solubility to water, which is more important for small molecules. Different binding mechanisms also explain the deeper penetration of etidocaine and TPP into the membrane core than calcium ions.

The relatively weak binding of metal cations (also other than sodium and calcium^75^) with a distinct mechanism to PC membranes suggests that they probably do not interfere with the binding of charged drugs with higher affinities, such as etidocaine, dibucaine, and SMS. The situation may be more complex for molecules with similar binding affinities but different mechanisms, such as calcium and TPP. While MD simulations are a promising tool to model such complicated systems, the accuracy of current force fields is not sufficient for such applications as none of the available force fields correctly captures both the calcium and charged small-molecule binding to membranes.

The evaluation of charged small-molecule binding affinity to membranes using changes in the lipid head-group-order parameters offers a tool to evaluate and develop force fields that would correctly capture interactions between charged biomolecules and membranes. This approach is complementary to the comparisons of partition coefficients^13,15^ as it gives information also on the detailed interactions between lipids and small molecules in addition to the binding affinity. While straightforward application of ECC to standard force fields has substantially improved the ion binding behavior,^18,19^ the small molecules seem to require further optimization. Nevertheless, we believe that our results pave the way toward force fields that would correctly capture lipid membrane interactions with charged biomolecules and amino acids. Such force fields have potential applications in a wide range of fields, from drug design to molecular biology and toxicology.

## Supporting Information Available

Force field parameters for TPP and etidocaine molecule, binding coefficients from the literature, additional analyses, and simulation details including a list of all simulations (PDF)

## References

[ref1] Lemmon M. A. Membrane recognition by phospholipid-binding domains. Nat. Rev. Mol. Cell Biol. 2008, 9, 99–111. 10.1038/nrm2328. 18216767

[ref2] Seddon A. M.; Casey D.; Law R. V.; Gee A.; Templer R. H.; Ces O. Drug interactions with lipid membranes. Chem. Soc. Rev. 2009, 38, 2509–2519. 10.1039/b813853m. 19690732

[ref3] Bittermann K.; Linden L.; Goss K.-U. Screening tools for the bioconcentration potential of monovalent organic ions in fish. Environ. Sci.: Processes Impacts 2018, 20, 845–853. 10.1039/c8em00084k. 29714798

[ref4] Dodd A. N.; Kudla J.; Sanders D. The language of calcium signaling. Annu. Rev. Plant Biol. 2010, 61, 593–620. 10.1146/annurev-arplant-070109-104628. 20192754

[ref5] Crilly S. E.; Puthenveedu M. A. Compartmentalized GPCR signaling from intracellular membranes. J. Membr. Biol. 2021, 254, 259–271. 10.1007/s00232-020-00158-7. 33231722 PMC8141539

[ref6] Hasan R.; Zhang X. Ca2+ regulation of TRP ion channels. Int. J. Mol. Sci. 2018, 19, 1256 10.3390/ijms19041256. PMC5979445 29690581

[ref7] Dutta A.; Vreeken J.; Ghiringhelli L. M.; Bereau T. Data-driven equation for drug-membrane permeability across drugs and membranes. J. Chem. Phys. 2021, 154, 244114 10.1063/5.0053931. 34241352

[ref8] Garcia D. S.; Sjödin M.; Hellstrandh M.; Norinder U.; Nikiforova V.; Lindberg J.; Wincent E.; Bergman Å.; Cotgreave I.; Kos V. M. Cellular accumulation and lipid binding of perfluorinated alkylated substances (PFASs)–A comparison with lysosomotropic drugs. Chem.-Biol. Interact. 2018, 281, 1–10. 10.1016/j.cbi.2017.12.021. 29248446

[ref9] Endo S.; Escher B. I.; Goss K.-U. Capacities of Membrane Lipids to Accumulate Neutral Organic Chemicals. Environ. Sci. Technol. 2011, 45, 5912–5921. 10.1021/es200855w. 21671592

[ref10] Jiang Z.; Reilly J. Chromatography approaches for early screening of the phospholipidosis-inducing potential of pharmaceuticals. J. Pharm. Biomed. Sci. 2012, 61, 184–190. 10.1016/j.jpba.2011.11.033. 22200505

[ref11] Klamt A.; Huniar U.; Spycher S.; Keldenich J. COSMOmic: A Mechanistic Approach to the Calculation of Membrane–Water Partition Coefficients and Internal Distributions within Membranes and Micelles. J. Phys. Chem. B 2008, 112, 12148–12157. 10.1021/jp801736k. 18754634

[ref12] Jakobtorweihen S.; Zuniga A. C.; Ingram T.; Gerlach T.; Keil F. J.; Smirnova I. Predicting solute partitioning in lipid bilayers: Free energies and partition coefficients from molecular dynamics simulations and COSMOmic. J. Chem. Phys. 2014, 141, 045102 10.1063/1.4890877. 25084963

[ref13] Paloncýová M.; Fabre G.; DeVane R. H.; Trouillas P.; Berka K.; Otyepka M. Benchmarking of Force Fields for Molecule-Membrane Interactions. J. Chem. Theory Comput. 2014, 10, 4143 10.1021/ct500419b. 26588554

[ref14] Bittermann K.; Spycher S.; Endo S.; Pohler L.; Huniar U.; Goss K.-U.; Klamt A. Prediction of Phospholipid-Water Partition Coefficients of Ionic Organic Chemicals Using the Mechanistic Model COSMOmic. J. Phys. Chem. B 2014, 118, 14833–14842. 10.1021/jp509348a. 25459490

[ref15] Bittermann K.; Spycher S.; Goss K.-U. Comparison of different models predicting the phospholipid-membrane water partition coefficients of charged compounds. Chemosphere 2016, 144, 382–391. 10.1016/j.chemosphere.2015.08.065. 26383265

[ref16] Catte A.; Girych M.; Javanainen M.; Loison C.; Melcr J.; Miettinen M. S.; Monticelli L.; Määttä J.; Oganesyan V. S.; Ollila O. H. S.; et al. Molecular electrometer and binding of cations to phospholipid bilayers. Phys. Chem. Chem. Phys. 2016, 18, 32560–32569. 10.1039/c6cp04883h. 27874109

[ref17] Venable R. M.; Luo Y.; Gawrisch K.; Roux B.; Pastor R. W. Simulations of Anionic Lipid Membranes: Development of Interaction-Specific Ion Parameters and Validation Using NMR Data. J. Phys. Chem. B 2013, 117, 10183–10192. 10.1021/jp401512z. 23924441 PMC3813009

[ref18] Melcr J.; Martinez-Seara H.; Nencini R.; Kolafa J.; Jungwirth P.; Ollila O. H. S. Accurate Binding of Sodium and Calcium to a POPC Bilayer by Effective Inclusion of Electronic Polarization. J. Phys. Chem. B 2018, 122, 4546–4557. 10.1021/acs.jpcb.7b12510. 29608850

[ref19] Melcr J.; Ferreira T. M.; Jungwirth P.; Ollila O. H. S. Improved Cation Binding to Lipid Bilayers with Negatively Charged POPS by Effective Inclusion of Electronic Polarization. J. Chem. Theory Comput. 2020, 16, 738–748. 10.1021/acs.jctc.9b00824. 31762275

[ref20] Bacle A.; Buslaev P.; Garcia-Fandino R.; Favela-Rosales F.; Mendes Ferreira T.; Fuchs P. F. J.; Gushchin I.; Javanainen M.; Kiirikki A. M.; Madsen J. J.; et al. Inverse Conformational Selection in Lipid-Protein Binding. J. Am. Chem. Soc. 2021, 143, 13701–13709. 10.1021/jacs.1c05549. 34465095

[ref21] Kim S.; Patel D.; Park S.; Slusky J.; Klauda J.; Widmalm G.; Im W. Bilayer Properties of Lipid A from Various Gram-Negative Bacteria. Biophys. J. 2016, 111, 1750–1760. 10.1016/j.bpj.2016.09.001. 27760361 PMC5071556

[ref22] Han K.; Venable R. M.; Bryant A.-M.; Legacy C. J.; Shen R.; Li H.; Roux B.; Gericke A.; Pastor R. W. Graph-Theoretic Analysis of Monomethyl Phosphate Clustering in Ionic Solutions. J. Phys. Chem. B 2018, 122, 1484–1494. 10.1021/acs.jpcb.7b10730. 29293344 PMC6322214

[ref23] Wang Y.; Zhao T.; Wei D.; Strandberg E.; Ulrich A. S.; Ulmschneider J. P. How reliable are molecular dynamics simulations of membrane active antimicrobial peptides?. Biochim. Biophys. Acta, Biomembr. 2014, 1838, 2280–2288. 10.1016/j.bbamem.2014.04.009. 24747526

[ref24] Fox S. J.; Lakshminarayanan R.; Beuerman R. W.; Li J.; Verma C. S. Conformational Transitions of Melittin between Aqueous and Lipid Phases: Comparison of Simulations with Experiments. J. Phys. Chem. B 2018, 122, 8698–8705. 10.1021/acs.jpcb.8b06781. 30114909

[ref25] Mustafa G.; Nandekar P. P.; Mukherjee G.; Bruce N. J.; Wade R. C. The Effect of Force-Field Parameters on Cytochrome P450-Membrane Interactions: Structure and Dynamics. Sci. Rep. 2020, 10, 7284 10.1038/s41598-020-64129-7. 32350331 PMC7190701

[ref26] Wang L.; O’Mara M. L. Effect of the Force Field on Molecular Dynamics Simulations of the Multidrug Efflux Protein P-Glycoprotein. J. Chem. Theory Comput. 2021, 17, 6491–6508. 10.1021/acs.jctc.1c00414. 34506133

[ref27] Sandoval-Perez A.; Pluhackova K.; Böckmann R. A. Critical Comparison of Biomembrane Force Fields: Protein-Lipid Interactions at the Membrane Interface. J. Chem. Theory Comput. 2017, 13, 2310–2321. 10.1021/acs.jctc.7b00001. 28388089

[ref28] MacCallum J. L.; Bennett W. F. D.; Tieleman D. P. Distribution of Amino Acids in a Lipid Bilayer from Computer Simulations. Biophys. J. 2008, 94, 3393–3404. 10.1529/biophysj.107.112805. 18212019 PMC2292383

[ref29] Lockhart C.; Smith A. K.; Klimov D. K. Three Popular Force Fields Predict Consensus Mechanism of Amyloid β Peptide Binding to the Dimyristoylgylcerophosphocholine Bilayer. J. Chem. Inf. Model. 2020, 60, 2282–2293. 10.1021/acs.jcim.0c00096. 32176493

[ref30] Mahmood M. I.; Yamashita T. Influence of Lipid Bilayer on the GPCR Structure: Comparison of All-Atom Lipid Force Fields. Bull. Chem. Soc. Jpn. 2021, 94, 2569–2574. 10.1246/bcsj.20210244.

[ref31] Reißer S.; Strandberg E.; Steinbrecher T.; Elstner M.; Ulrich A. S. Best of Two Worlds? How MD Simulations of Amphiphilic Helical Peptides in Membranes Can Complement Data from Oriented Solid-State NMR. J. Chem. Theory Comput. 2018, 14, 6002–6014. 10.1021/acs.jctc.8b00283. 30289704

[ref32] Johansson A. C. V.; Lindahl E. Position-resolved free energy of solvation for amino acids in lipid membranes from molecular dynamics simulations. Proteins: Struct., Funct., Genet. 2008, 70, 1332–1344. 10.1002/prot.21916. 17876818

[ref33] Antila H.; Buslaev P.; Favela-Rosales F.; Ferreira T. M.; Gushchin I.; Javanainen M.; Kav B.; Madsen J. J.; Melcr J.; Miettinen M. S.; et al. Headgroup Structure and Cation Binding in Phosphatidylserine Lipid Bilayers. J. Phys. Chem. B 2019, 123, 9066–9079. 10.1021/acs.jpcb.9b06091. 31574222

[ref34] Seelig J.; MacDonald P. M.; Scherer P. G. Phospholipid head groups as sensors of electric charge in membranes. Biochemistry 1987, 26, 7535–7541. 10.1021/bi00398a001. 3322401

[ref35] Clarke R. J. The dipole potential of phospholipid membranes and methods for its detection. Adv. Colloid Interface Sci. 2001, 89-90, 263–281. 10.1016/s0001-8686(00)00061-0. 11215797

[ref36] Klauda J. B.; Venable R. M.; Freites J. A.; O’Connor J. W.; Tobias D. J.; Mondragon-Ramirez C.; Vorobyov I.; MacKerell A. D.; Pastor R. W. Update of the CHARMM all-atom additive force field for lipids: validation on six lipid types. J. Phys. Chem. B 2010, 114, 7830–7843. 10.1021/jp101759q. 20496934 PMC2922408

[ref37] Jo S.; Kim T.; Iyer V. G.; Im W. CHARMM-GUI: a web-based graphical user interface for CHARMM. J. Comput. Chem. 2008, 29, 1859–1865. 10.1002/jcc.20945. 18351591

[ref38] Brooks B. R.; Brooks C. L.; Mackerell A. D.; Nilsson L.; Petrella R. J.; Roux B.; Won Y.; Archontis G.; Bartels C.; Boresch S.; et al. CHARMM: the biomolecular simulation program. J. Comput. Chem. 2009, 30, 1545–1614. 10.1002/jcc.21287. 19444816 PMC2810661

[ref39] Lee J.; Cheng X.; Swails J. M.; Yeom M. S.; Eastman P. K.; Lemkul J. A.; Wei S.; Buckner J.; Jeong J. C.; Qi Y.; et al. CHARMM-GUI input generator for NAMD, GROMACS, AMBER, OpenMM, and CHARMM/OpenMM simulations using the CHARMM36 additive force field. J. Chem. Theory Comput. 2016, 12, 405–413. 10.1021/acs.jctc.5b00935. 26631602 PMC4712441

[ref40] Zoete V.; Cuendet M. A.; Grosdidier A.; Michielin O. SwissParam: a fast force field generation tool for small organic molecules. J. Comput. Chem. 2011, 32, 2359–2368. 10.1002/jcc.21816. 21541964

[ref41] Vanommeslaeghe K.; Hatcher E.; Acharya C.; Kundu S.; Zhong S.; Shim J.; Darian E.; Guvench O.; Lopes P.; Vorobyov I.; et al. CHARMM general force field: A force field for drug-like molecules compatible with the CHARMM all-atom additive biological force fields. J. Comput. Chem. 2010, 31, 671 10.1002/jcc.21367. 19575467 PMC2888302

[ref42] Vanommeslaeghe K.; MacKerell A. D. Jr. Automation of the CHARMM General Force Field (CGenFF) I: bond perception and atom typing. J. Chem. Inf. Model. 2012, 52, 3144–3154. 10.1021/ci300363c. 23146088 PMC3528824

[ref43] Vanommeslaeghe K.; Raman E. P.; MacKerell A. D. Jr. Automation of the CHARMM General Force Field (CGenFF) II: assignment of bonded parameters and partial atomic charges. J. Chem. Inf. Model. 2012, 52, 3155–3168. 10.1021/ci3003649. 23145473 PMC3528813

[ref44] Breneman C. M.; Wiberg K. B. Determining atom-centered monopoles from molecular electrostatic potentials. The need for high sampling density in formamide conformational analysis. J. Comput. Chem. 1990, 11, 361–373. 10.1002/jcc.540110311.

[ref45] Schamberger J.; Clarke R. J. Hydrophobic ion hydration and the magnitude of the dipole potential. Biophys. J. 2002, 82, 3081–3088. 10.1016/s0006-3495(02)75649-x. 12023231 PMC1302096

[ref46] MacKerell A. D. Jr.; Bashford D.; Bellott M.; Dunbrack R. L. Jr.; Evanseck J. D.; Field M. J.; Fischer S.; Gao J.; Guo H.; Ha S.; et al. All-atom empirical potential for molecular modeling and dynamics studies of proteins. J. Phys. Chem. B 1998, 102, 3586–3616. 10.1021/jp973084f. 24889800

[ref47] Nencini R. Development and testing of computer models of phospholipid membranes. MSc Thesis, Charles University of Prague, 2019.

[ref48] Leontyev I.; Stuchebrukhov A. Accounting for electronic polarization in non-polarizable force fields. Phys. Chem. Chem. Phys. 2011, 13, 2613–2626. 10.1039/c0cp01971b. 21212894

[ref49] Duboué-Dijon E.; Javanainen M.; Delcroix P.; Jungwirth P.; Martinez-Seara H. A practical guide to biologically relevant molecular simulations with charge scaling for electronic polarization. J. Chem. Phys. 2020, 153, 050901 10.1063/5.0017775. 32770904

[ref50] Abraham M. J.; Murtola T.; Schulz R.; Páll S.; Smith J. C.; Hess B.; Lindahl E. GROMACS: High performance molecular simulations through multi-level parallelism from laptops to supercomputers. SoftwareX 2015, 1-2, 19–25. 10.1016/j.softx.2015.06.001.

[ref51] Páll S.; Zhmurov A.; Bauer P.; Abraham M.; Lundborg M.; Gray A.; Hess B.; Lindahl E. Heterogeneous parallelization and acceleration of molecular dynamics simulations in GROMACS. J. Chem. Phys. 2020, 153, 134110 10.1063/5.0018516. 33032406

[ref52] Nosé S. A unified formulation of the constant temperature molecular dynamics methods. J. Chem. Phys. 1984, 81, 511–519. 10.1063/1.447334.

[ref53] Hoover W. G. Canonical dynamics: Equilibrium phase-space distributions. Phys. Rev. A 1985, 31, 1695 10.1103/physreva.31.1695. 9895674

[ref54] Darden T.; York D.; Pedersen L. Particle mesh Ewald: AnN log(N) method for Ewald sums in large systems. J. Chem. Phys. 1993, 98, 10089–10092. 10.1063/1.464397.

[ref55] Essmann U.; Perera L.; Berkowitz M. L.; Darden T.; Lee H.; Pedersen L. G. A smooth particle mesh Ewald method. J. Chem. Phys. 1995, 103, 8577–8593. 10.1063/1.470117.

[ref56] Parrinello M.; Rahman A. Polymorphic transitions in single crystals: A new molecular dynamics method. J. Appl. Phys. 1981, 52, 7182–7190. 10.1063/1.328693.

[ref57] Gowers R. J.; Linke M.; Barnoud J.; Reddy T. J. E.; Melo M. N.; Seyler S. L.; Domański J.; Dotson D. L.; Buchoux S.; Kenney I. M.; MDAnalysis: a Python package for the rapid analysis of molecular dynamics simulations. Proceedings of the Python in Science Conference, Proceedings of the 15th Python in Science Conference, 2016.

[ref58] Michaud-Agrawal N.; Denning E. J.; Woolf T. B.; Beckstein O. MDAnalysis: a toolkit for the analysis of molecular dynamics simulations. J. Comput. Chem. 2011, 32, 2319–2327. 10.1002/jcc.21787. 21500218 PMC3144279

[ref59] de Souza Camilo C. R.; Ruggiero J. R.; de Araujo A. S. A Method for Detection of Water Permeation Events in Molecular Dynamics Simulations of Lipid Bilayers. Braz. J. Phys. 2022, 52, 62 10.1007/s13538-022-01071-1.

[ref60] Jämbeck J. P. M.; Lyubartsev A. P. Implicit inclusion of atomic polarization in modeling of partitioning between water and lipid bilayers. Phys. Chem. Chem. Phys. 2013, 15, 4677–4686. 10.1039/c3cp44472d. 23439978

[ref61] Marsh D. Handbook of Lipid Bilayers, 2nd ed.; CRC Press, 2013.

[ref62] Beschiaschvili G.; Seelig J. Peptide binding to lipid bilayers. Binding isotherms and .zeta.-potential of a cyclic somatostatin analog. Biochemistry 1990, 29, 10995–11000. 10.1021/bi00501a018. 2271694

[ref63] Altenbach C.; Seelig J. Binding of the lipophilic cation tetraphenylphosphonium to phosphatidylcholine membranes. Biochim. Biophys. Acta, Biomembr. 1985, 818, 410–415. 10.1016/0005-2736(85)90016-1.

[ref64] Seelig A.; Allegrini P. R.; Seelig J. Partitioning of local anesthetics into membranes: surface charge effects monitored by the phospholipid head-group. Biochim. Biophys. Acta, Biomembr. 1988, 939, 267–276. 10.1016/0005-2736(88)90070-3. 3355817

[ref65] Altenbach C.; Seelig J. Calcium binding to phosphatidylcholine bilayers as studied by deuterium magnetic resonance. Evidence for the formation of a calcium complex with two phospholipid molecules. Biochemistry 1984, 23, 3913–3920. 10.1021/bi00312a019. 6487586

[ref66] Beschiaschvili G.; Seelig J. Peptide binding to lipid membranes. Spectroscopic studies on the insertion of a cyclic somatostatin analog into phospholipid bilayers. Biochim. Biophys. Acta, Biomembr. 1991, 1061, 78–84. 10.1016/0005-2736(91)90270-i. 1995058

[ref67] Lis L. J.; Parsegian V. A.; Rand R. P. Binding of divalent cations to dipalmitoylphosphatidylcholine bilayers and its effect on bilayer interaction. Biochemistry 1981, 20, 1761–1770. 10.1021/bi00510a009. 6164391

[ref68] Li H.; Chowdhary J.; Huang L.; He X.; MacKerell A. D. Jr.; Roux B. Drude polarizable force field for molecular dynamics simulations of saturated and unsaturated zwitterionic lipids. J. Chem. Theory Comput. 2017, 13, 4535–4552. 10.1021/acs.jctc.7b00262. 28731702 PMC5595662

[ref69] Antila H. S.; Kav B.; Miettinen M. S.; Martinez-Seara H.; Jungwirth P.; Ollila O. H. S. Emerging Era of Biomolecular Membrane Simulations: Automated Physically-Justified Force Field Development and Quality-Evaluated Databanks. J. Phys. Chem. B 2022, 126, 4169–4183. 10.1021/acs.jpcb.2c01954.

[ref70] Nencini R. Development of PROSECCO PC membranes - PN model, POPC membranes, different calcium and sodium concentrations, 310K. 2019; Online, https://doi.org/10.5281/zenodo.3336193 (accessed Jan 29, 2022).

[ref71] Nencini R. CHARMM36, NB-Fix approaches, without NBFIX, POPC membrane, Ca, Na ions. 2019; Online, https://doi.org/10.5281/zenodo.3434396 (accessed Jan 29, 2022).

[ref72] Melcr J. Simulations of POPC lipid bilayer in water solution at various NaCl, KCl and CaCl2 concentrations using ECC-POPC force field. 2017; Online, https://doi.org/10.5281/zenodo.3335503 (accessed Jan 29, 2022).

[ref73] Melcr J. Simulations of a POPC lipid bilayer in water solution at various NaCl and CaCl2 concentration with Lipid14, TIP3p and Dang or ECC ions. 2017; Online, https://doi.org/10.5281/zenodo.1111822 (accessed Jan 29, 2022).

[ref74] Martinez-Seara H. POPC bilayer, 154 mM NaCl ions, prosECCo75 version of CHARMM36 FF, 310K, gromacs 2021.2. 2022; Online, https://doi.org/10.5281/zenodo.6535604 (accessed Jan 29, 2022).

[ref75] Binder H.; Zschörnig O. The effect of metal cations on the phase behavior and hydration characteristics of phospholipid membranes. Chem. Phys. Lipids 2002, 115, 39–61. 10.1016/s0009-3084(02)00005-1. 12047897

