# Supplementary material for: Charged Small Molecule Binding to Membranes in MD Simulations Evaluated against NMR Experiments
Source: J Phys Chem B. 2022 Sep 5;126(36):6955–63. doi: 10.1021/acs.jpcb.2c05024 (PMC9483918; doi:10.1021/acs.jpcb.2c05024)
Supplement: Supplementary file 1 — jp2c05024_si_001.pdf [file jp2c05024_si_001.pdf]

Supporting Information for: Charged Small Molecule Binding to Membranes in MD Simulations Evaluated Against NMR Experiments

Ricky Nencini and O. H. Samuli Ollila*

Insititute of Biotechnology, University of Helsinki, 00014 University of Helsinki, Finland

E-mail: samuli.ollila@helsinki.fi

Partial charges of TPP

a)

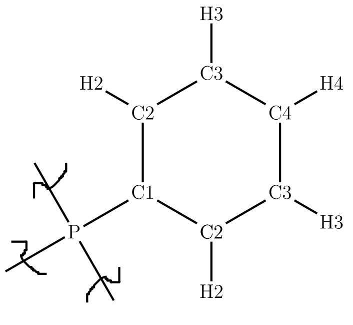

b)

Atom	CHARMM36 Protein	B3LYP	B3LYP ECC	Clarke	Clarke ECC
P	1.000	0.184	0.13800	0.728	0.546
C1	0.000	0.044	0.03300	-0.111	-0.083
C2	-0.115	-0.047	-0.03525	-0.170	-0.128
C3	-0.115	-0.091	-0.06825	-0.150	-0.113
C4	-0.115	-0.053	-0.03975	-0.143	-0.107
H2	0.115	0.061	0.04575	0.199	0.150
H3	0.115	0.125	0.09375	0.188	0.141
H4	0.115	0.117	0.08775	0.188	0.141

Figure S1: a) Chemical structure and atom names of tetraphenylphosphonium with four identical phenyl rings. For simplicity only one ring is shown b) Partial charges obtained from phenyl ring in phenyl-alanine in protein CHARMM36 force field (CHARMM36 Protein), calculated with Gaussian using B3LYP functional, 6-31G** basis set (B3LYP), obtained from literature[1] (Clarke), and the respective ECC variants (B3LYP ECC and Clarke ECC).

Partial charges of etidocaine

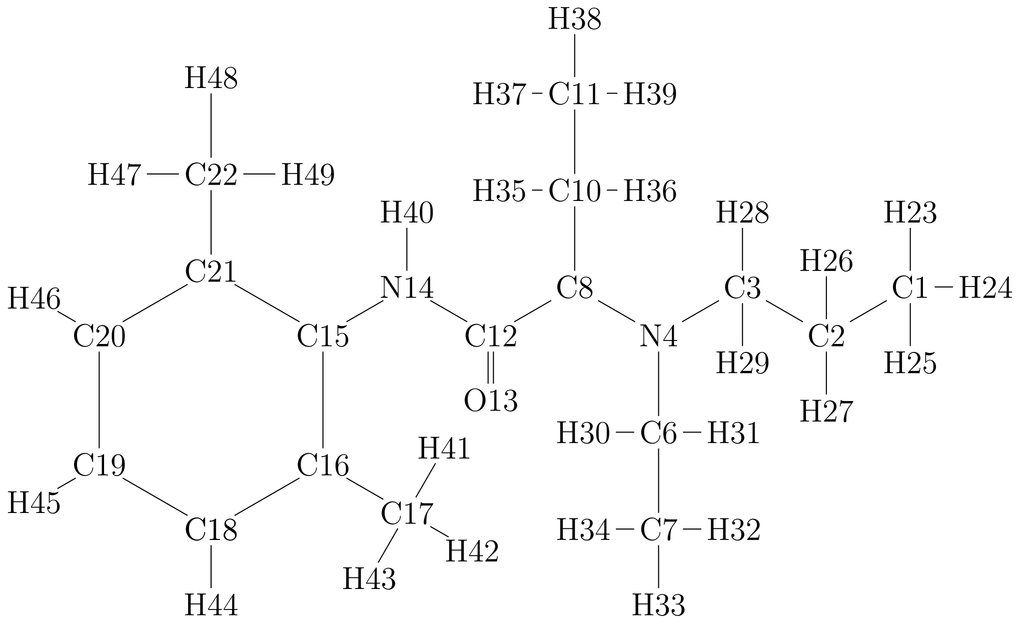

Figure S2: Chemical structure and atom names of etidocaine.

Table S1: Partial charges of etidocaine from SwissParam and Cgenff (ParamChem), and after applying ECC for Cgenff parameters.

**Atom**	**SwissParam**	**ParamChem**	**ParamChem ECC**
C15	0.1170	0.191	0.14325
C16	-0.1435	-0.010	-0.00750
C18	-0.1500	-0.113	-0.08475
C19	-0.1500	-0.115	-0.08625
C20	-0.1500	-0.113	-0.08475
C21	-0.1435	-0.010	-0.00750
C1	0.0000	-0.272	-0.20400
C2	0.0000	-0.177	-0.13275
C3	0.5030	0.121	0.09075
N4	-0.9590	-0.351	-0.26325
H5	0.4500	0.320	0.24000
C6	0.5030	0.127	0.09525
C7	0.0000	-0.268	-0.20100
C8	0.5640	0.397	0.29775
H9	0.0000	0.090	0.06750
C10	0.0000	-0.220	-0.16500
C11	0.0000	-0.256	-0.19200
C12	0.5690	0.373	0.27975
O13	-0.5700	-0.490	-0.36750
N14	-0.5470	-0.423	-0.31725
C17	0.1435	-0.267	-0.20025
C22	0.1435	-0.267	-0.20025
H23	0.0000	0.090	0.06750
H24	0.0000	0.090	0.06750
H25	0.0000	0.090	0.06750
H26	0.0000	0.090	0.06750
H27	0.0000	0.090	0.06750
H28	0.0000	0.090	0.06750
H29	0.0000	0.090	0.06750
H30	0.0000	0.090	0.06750
H31	0.0000	0.090	0.06750
H32	0.0000	0.090	0.06750
H33	0.0000	0.090	0.06750
H34	0.0000	0.090	0.06750
H35	0.0000	0.090	0.06750
H36	0.0000	0.090	0.06750
H37	0.0000	0.090	0.06750
H38	0.0000	0.090	0.06750
H39	0.0000	0.090	0.06750
H40	0.3700	0.318	0.23850
H41	0.0000	0.090	0.06750
H42	0.0000	0.090	0.06750
H43	0.0000	0.090	0.06750
H44	0.1500	0.115	0.08625
H45	0.1500	0.115	0.08625
H46	0.1500	0.115	0.08625
H47	0.0000	0.090	0.06750
H48	0.0000	0.090	0.06750
H49	0.0000	0.090	0.06750

Calculation of the number of bound molecules

Defining the number of bound molecules to membranes is not straightforward in MD simulations.[2] Nevertheless, the number of simple ions in the vicinity of lipid oxygens seems to be practically independent on the selected cut-off approximately between 0.25-0.45,[3] thereby being probably a reasonable measure for the number of bound ions. The case of molecules with multiple atoms is more complicated because also the minimum number of small molecule-lipid contacts required for the interpreted binding needs to be defined. Inspired by the approach used for simple ions,[3] we first calculated the number of bound molecules as a function of cut-off value and the required number of contacts (Fig. S3 a), and then calculated the point of smallest divergence with non-zero value from this 3D graph (Fig. S3 b). At this point, the result is least dependent on the definition of bound molecules, and its value was therefore used as the definition for the number of bound molecules.

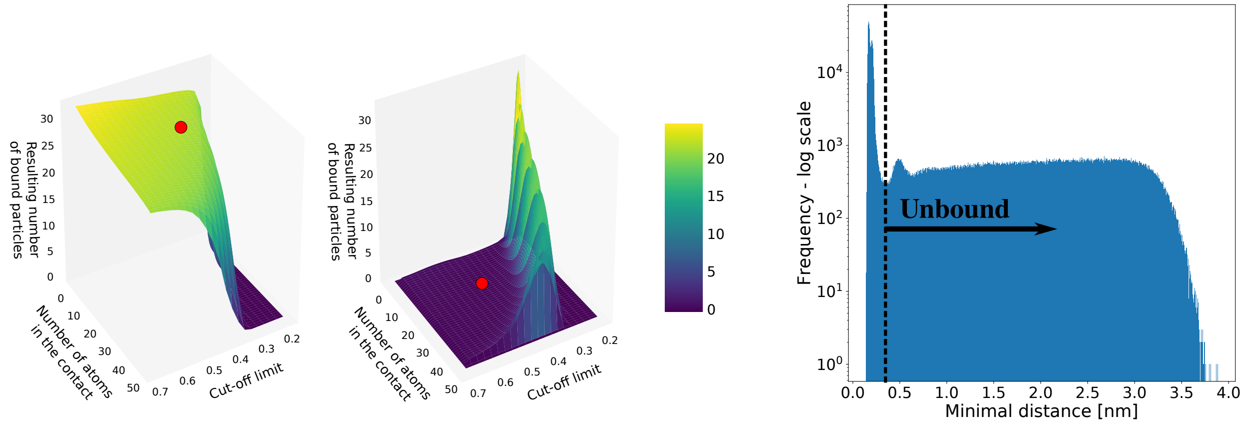

Figure S3: **a):** Number of bound particles as a function of cut-off distance and the required minimum number of particle's atoms in contact with lipids for a simulation with 140 mM Etidocaine and repeat distance of 12 nm. **b):** Divergence of the data in a). The point with smallest divergence with non-zero number of bound molecules is used as the definition for the number of bound particles. **c):** Histogram of minimal distances between any etidocaine and lipid atom as analyzed in.[4]

Alternative approach used to determine the number of bound molecules with multiple atoms to membranes is based on the histogram of minimum distances between any lipid atom and any atom of the particle of interest.[4] The histogram is characterized by the peak

at small distances followed by a minima, found at 0.45 nm in Fig. S3c, and the particles with the minimum distance below this value are then defined to be bound. Resulting numbers of bound molecules are comparable between the two definitions.

Binding coefficients from the literature

Table S2: Binding coefficients of different molecules reported in the literature determined from NMR data by assuming a model for the binding to lipids.

**Binding Particle**	**Reference**	**Reported Binding Constant**	**Model Used**
Ca, no NaCl	`https://doi.org/10.1021/bi00312a019`	7.0 M^{-1}	ternary complex
Ca, 0.1M NaCl	`https://doi.org/10.1021/bi00312a019`	13.7 M^{-1}	ternary complex
TPP, 0.1M NaCl	`https://doi.org/10.1016/0005-2736(85)90016-1`	21 M^{-2}	Langmuir 1:8.3
Eticocaine, 0.1M NaCl	`https://doi.org/10.1016/0005-2736(88)90070-3`	11 M^{-1}	Partition eq.
Dibucaine, 0.1M NaCl	`https://doi.org/10.1016/0005-2736(88)90070-3`	660 M^{-1}	Partition eq.
SMS, 0.1M NaCl	`https://doi.org/10.1021/bi00501a018`	66 M^{-1}	Partition eq. + Gouy-Chapman

Dependence on simulation box size

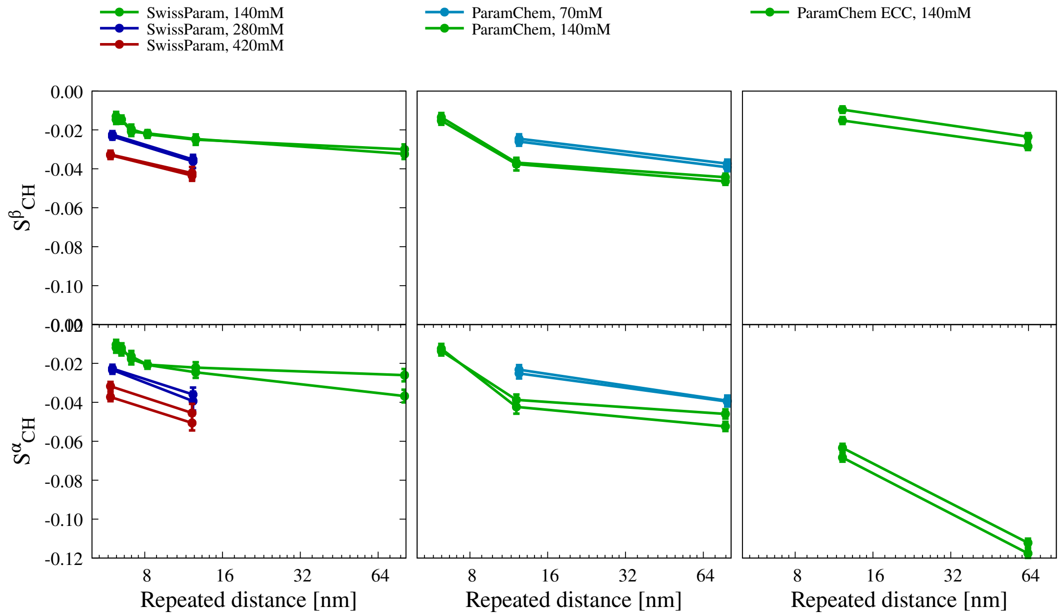

Figure S4: Dependence of the POPC headgroup order parameters on the simulation box size in the membrane normal direction (repeat distance) with different etidocaine concentrations and different force fields (left: CHARMM36-SwissParam, middle: CHARMM36-ParamChem, right: CHARMM36-ParamChemECC).

Equilibration of the simulations

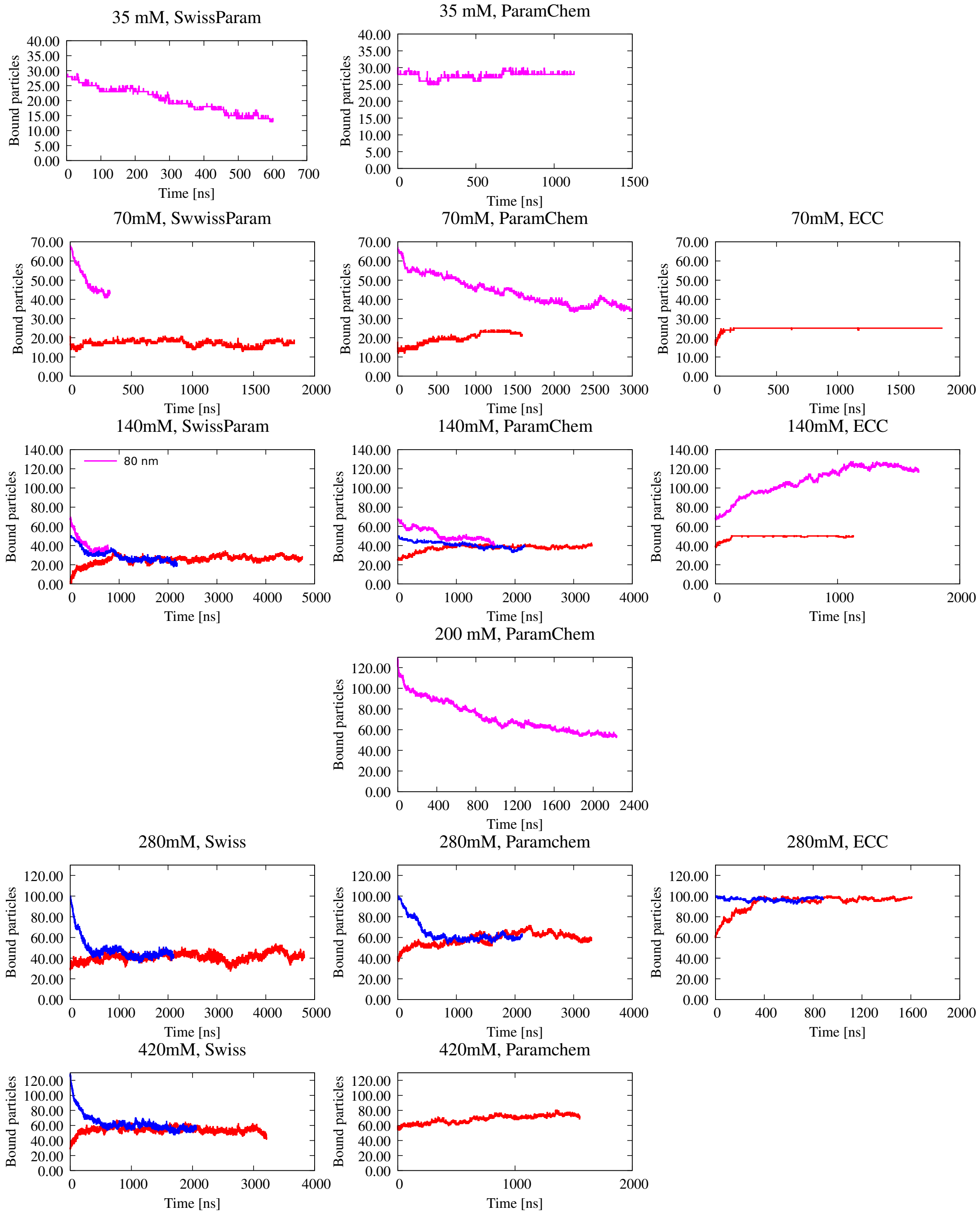

Figure S5: **Binding of Etidocaine, ETI, in time** Left column: systems run with SwissParam force field. Middle column: systems run with ParamChem force field. Right column: systems run with ECC version of ParamChem force field. In red: systems with approximately 20 000 water molecules (repeat distance ∼12 nm) started from unsaturated binding state. In blue: systems with approximately 20 000 water molecules started from over-saturated binding state. In magenta: systems simulated with 177 600 water molecules (repeat distance ∼12 nm).

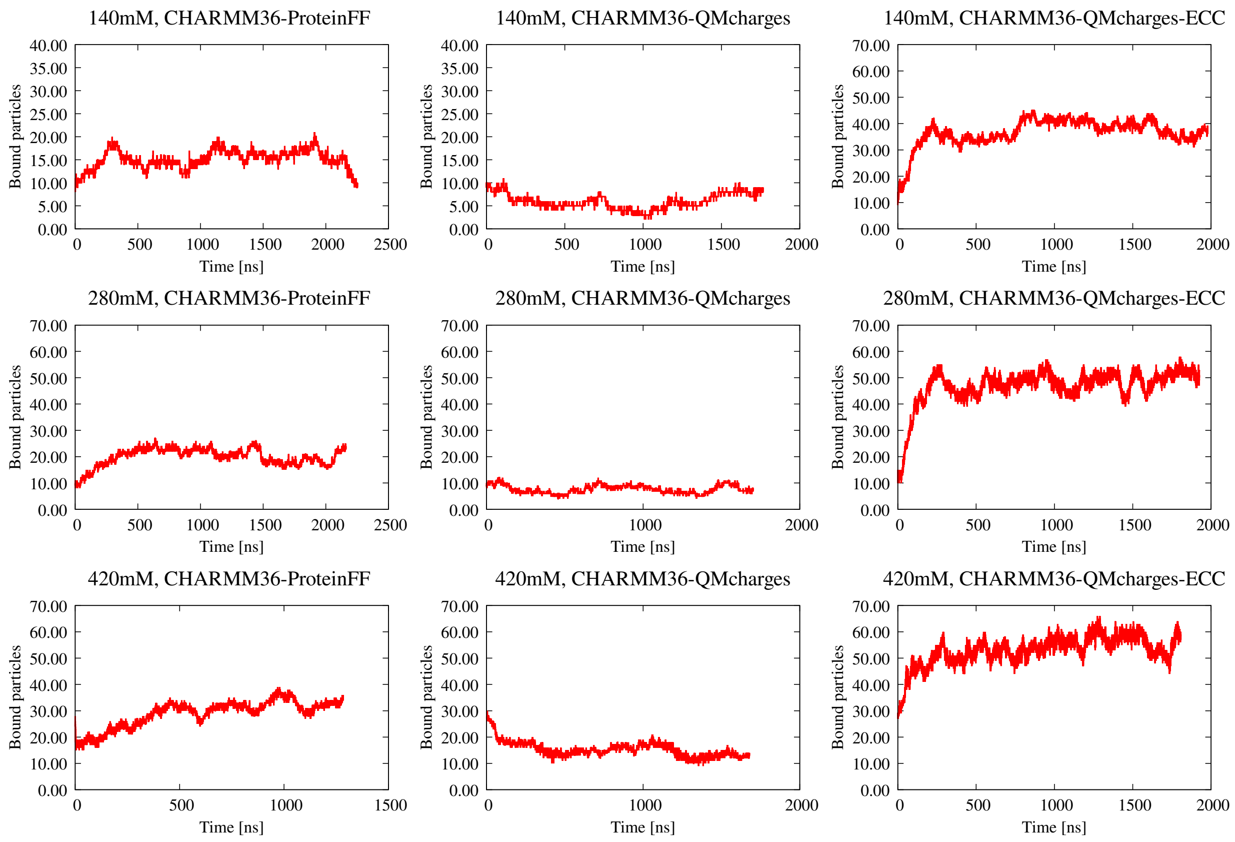

Figure S6: Number of bound TPP to POPC membrane as a function of time at different concentrations from simulations with different force field parameters used in the main text: CHARMM36-ProteinFF (left), CHARMM36-QMcharges (middle) and CHARMM36-QMcharges-ECC (right).

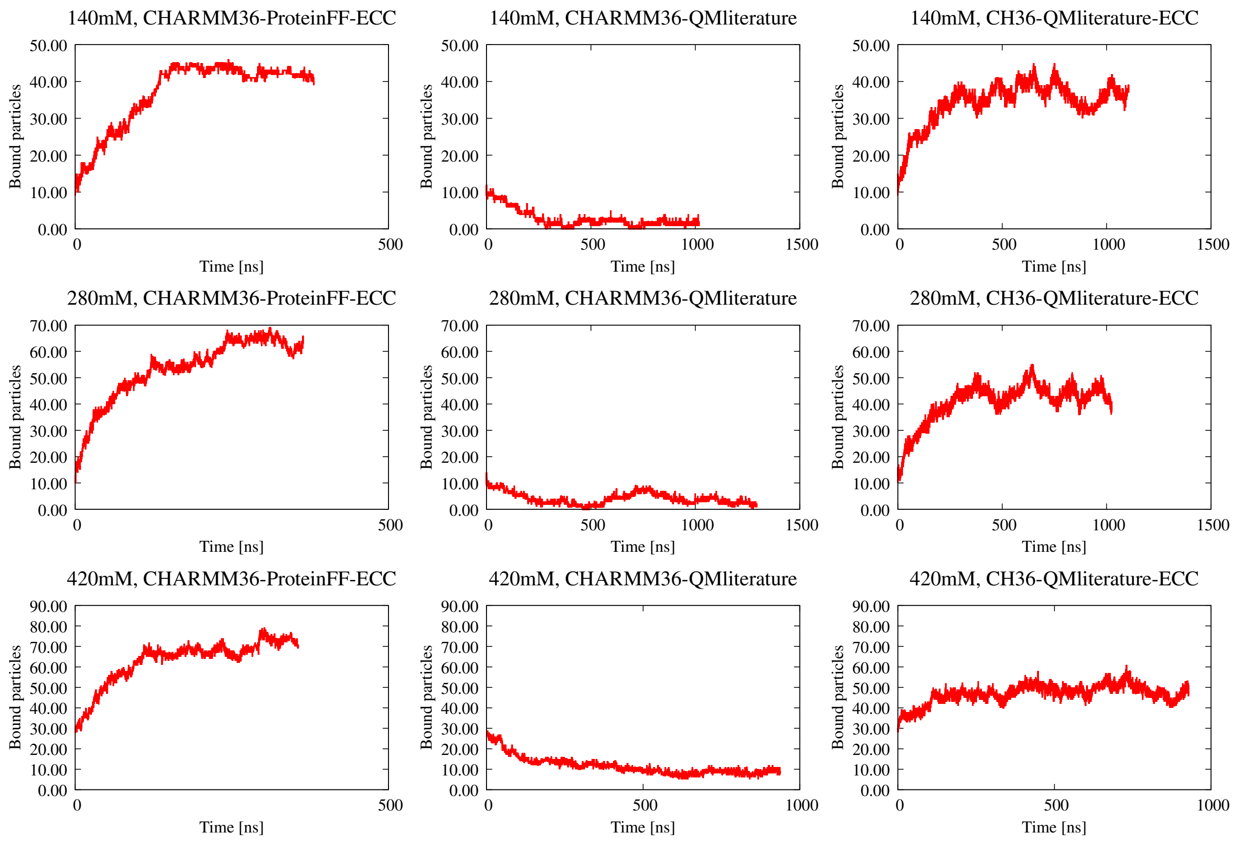

Figure S7: Number of bound TPP to POPC membrane as a function of time at different concentrations from simulations with different force field parameters that were not used in the main text: CHARMM36-ProteinFF with an ECC (left), CHARMM36 force field with charges taken from literature[5] (middle) and ECC applied to the charges from the literature[5] (right).

Density profiles

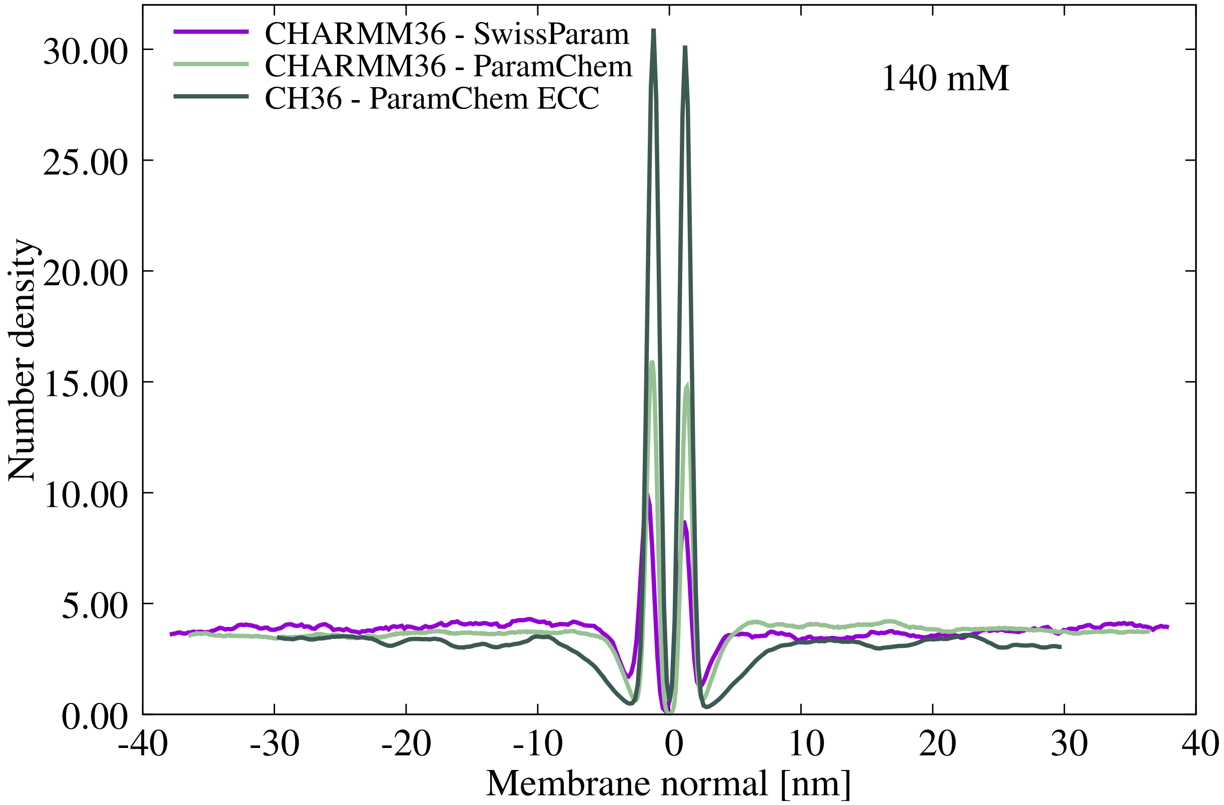

Figure S8: Molecule number density profiles of Etidocaine from simulations with POPC bilayer using different force field parameters. Profiles are calculated from systems with large box containing 177600 water molecules and 140 mM of etidocaine.

Simulated systems

Etidocaine

Table S3: Simulated POPC bilayer system with etidocaine using CHARMM36-SwissParam parameters. All systems contain 200 lipids, have Cl^- counter ions, and were simulated at at 298 K.

Etidocaine concentration[a] [mM]	Length [ns]	Equil.[b] [ns]	ETI[c]/ Cl^- [c]	Waters[c]	Repeat distance[d] [nm]	APL[d] [nm^2]	Ref.[e]
69	1 838	200	25	19 900	12.7	0.67	6
138	4 744	800	50	20 000	12.6	0.69	7
138[e]	2 183	1000	50	20 000	12.6	0.68	8
138	1 142	414	444	177 600	80.7	0.71	9
280	5 092	900	100	19 800	12.3	0.72	10
280[e]	2 114	500	100	19 800	12.2	0.72	11
418	3 212	500	150	19 872	12.2	0.74	12
418[e]	2 057	1000	150	19 872	12.2	0.75	13
12[f]	299	200	2	8 873	8.0	0.64	—
36[f]	601	601	111	166 608	—	—	—
69[f]	324	324	222	177 600	—	—	—
139[f]	713	200	13	5 187	6.2	0.66	—
139[f]	1 123	400	15	5 970	6.5	0.67	—
139[f]	987	550	19	7 562	7.1	0.67	—
139[f]	1 754	200	25	9 950	8.2	0.68	—
278[f]	1 693	200	26	5 174	6.0	0.68	—
416[f]	1 345	200	39	5 200	5.9	0.71	—
555[f]	646	100	52	5 200	5.8	0.73	

a) Calculated as ETI/waters * 55.5 * 1000.
b) Beginning part of the simulation left out as equilibration.
c) Number of molecules in the system.
d) Analysed from the equilibrated part.
e) Excessive number of etidocaine molecules was bound at the beginning.
f) Used for equlibration and box size experiments.
e) The number of reference where simulation files are available.

Table S4: Simulated POPC bilayer system with etidocaine using CHARMM36-ParamChem parameters. All systems contain 200 lipids, have Cl^- counter ions, and were simulated at at 298 K.

**Etidocaine concentration**[a] **[mM]**	**Length [ns]**	**Equil.**[b] **[ns]**	**ETI**[c]**/ Cl^-**[c]	**Waters**[c]	**Repeat distance**[d] **[nm]**	**APL**[d] **[nm^2]**	**Ref.**[g]
34	1 267	200	111	177 600	80.4	0.69	14
69	2 988	2 200	222	177 600	79.2	0.71	15
138	1 648	730	444	177 600	77.9	0.74	16
200	2 599	2 000	640	177 600	77.5	0.75	17
69[f]	1 596	1 100	25	19 900	12.4	0.68	—
139[f]	667	0	13	5 187	6.2	0.66	—
138[f]	3 628	500	50	20 000	12.1	0.72	—
138[e,f]	2 167	1 300	50	20 000	12.1	0.71	—
138[f]	588	588	444	177 600	—	—	—
278[f]	664	200	26	5 174	6.0	0.68	—
280[f]	3 610	1 323	100	19 800	11.5	0.76	—
280[e,f]	2 123	500	100	19 800	11.5	0.76	—
416[f]	656	50	39	5 200	5.9	0.71	—
418[f]	3 552	800	150	19 872	11.5	0.78	—
418[e,f]	2 083	500	150	19 872	11.5	0.79	—
1972[f]	669	669	400	11 252	—	—	—

a) Calculated as ETI/waters * 55.5 * 1000.

b) Beginning part of the simulation left out as equilibration.

c) Number of molecules in the system.

d) Analysed from the equilibrated part.

e) Excessive number of etidocaine molecules was bound at the beginning.

f) Used for equlibration and box size experiments.

g) The number of reference where simulation files are available.

Table S5: Simulated POPC bilayer system with etidocaine using CHARMM36-ParamChem-ECC parameters. All systems contain 200 lipids, have Cl^- counter ions, and were simulated at at 298 K.

**Etidocaine concentration**[a] **[mM]**	**Length [ns]**	**Equil.**[b] **[ns]**	**ETI**[c]**/ Cl^-** [c]	**Waters**[c]	**Repeat distance**[d] **[nm]**	**APL**[d] **[nm^2]**	**Ref.**[g]
138	1 664	1 100	444	177 600	63.7	0.9	18
69[f]	1 859	200	25	19 900	13.0	0.65	—
138[f]	1 132	200	50	20 000	12.2	0.71	—
280[f]	1 609	500	100	19 800	10.7	0.83	—
280[e,f]	881	50	100	19 800	10.7	0.83	—

a) Calculated as ETI/waters * 55.5 * 1000.

b) Beginning part of the simulation left out as equilibration.

c) Number of molecules in the system.

d) Analysed from the equilibrated part.

e) Excessive number of etidocaine molecules was bound at the beginning.

f) Used for equlibration and box size experiments.

g) The number of reference where simulation files are available.

Tetraphenylphosphonium ion

Table S6: Simulated POPC bilayer system with tetraphenylphosphonium ions using CHARMM36-ProteinFF parameters. All systems contain 200 lipids, have Cl^- counter ions, and were simulated at 298 K.

**TPP concentration**[a] **[mM]**	**Length [ns]**	**Equil.**[b] **[ns]**	**TPP**[c]**/ Cl^-**[c]	**Waters**[c]	**Repeat distance**[d] **[nm]**	**APL**[d] **[nm^2]**	**Ref.**[f]
138	2 283	200	50	20 000	12.9	0.67	19
277	2 194	500	100	20 000	13.1	0.68	20
416	2 000	500	150	20 000	13.1	0.70	21
106[e]	672	0	10	5 210	6.2	0.65	—
212[e]	323	130	20	5 220	6.2	0.67	—
100[e]	563	400	20	11 041	8.9	0.66	—

a) Calculated as TPP/waters * 55.5 * 1000.
b) Beginning part of the simulation left out as equilibration.
c) Number of molecules in the system.
d) Analysed from the equilibrated part.
e) Used for equlibration and box size experiments.
f) The number of reference where simulation files are available.

Table S7: Simulated POPC bilayer system with tetraphenylphosphonium ions using CHARMM36-ProteinFF-ECC parameters. All systems contain 200 lipids, have Cl^- counter ions, and were simulated at 298 K.

**TPP concentration**[a] **[mM]**	**Length [ns]**	**Equil.**[b] **[ns]**	**TPP**[c]**/ Cl^-**[c]	**Waters**[c]	**Repeat distance**[d] **[nm]**	**APL**[d] **[nm^2]**	**Ref.**[e]
138	1 039	160	50	20 000	12.3	0.71	22
277	1 007	250	100	20 000	11.8	0.76	23
416	994	300	150	20 000	12.0	0.77	24

a) Calculated as TPP/waters * 55.5 * 1000.
b) Beginning part of the simulation left out as equilibration.
c) Number of molecules in the system.
d) Analysed from the equilibrated part.
e) The number of reference where simulation files are available.

Table S8: Simulated POPC bilayer system with tetraphenylphosphonium ions using CHARMM36-QMcharges parameters. All systems contain 200 lipids, have Cl^- counter ions, and were simulated at 298 K.

**TPP concentration**[a] **[mM]**	**Length [ns]**	**Equil.**[b] **[ns]**	**TPP**[c]**/ Cl^-** [c]	**Waters**[c]	**Repeat distance**[d] **[nm]**	**APL**[d] **[nm^2]**	**Ref.**[e]
138	2 000	200	50	20 000	13.3	0.65	25
277	2 000	400	100	20 000	13.6	0.66	26
416	1 679	400	150	20 000	13.8	0.67	27

a) Calculated as TPP/waters * 55.5 * 1000.
b) Beginning part of the simulation left out as equilibration.
c) Number of molecules in the system.
d) Analysed from the equilibrated part.
e) The number of reference where simulation files are available.

Table S9: Simulated POPC bilayer system with tetraphenylphosphonium ions using CHARMM36-QMcharges-ECC parameters. All systems contain 200 lipids, have Cl^- counter ions, and were simulated at 298 K.

**TPP concentration**[a] **[mM]**	**Length [ns]**	**Equil.**[b] **[ns]**	**TPP**[c]**/ Cl^-** [c]	**Waters**[c]	**Repeat distance**[d] **[nm]**	**APL**[d] **[nm^2]**	**Ref.**[e]
138	2 000	200	50	20 000	12.1	0.72	28
277	2 000	200	100	20 000	12.0	0.75	29
416	2 000	500	150	20 000	12.2	0.76	30

a) Calculated as TPP/waters * 55.5 * 1000.
b) Beginning part of the simulation left out as equilibration.
c) Number of molecules in the system.
d) Analysed from the equilibrated part.
e) The number of reference where simulation files are available.

Table S10: Simulated POPC bilayer system with tetraphenylphosphonium ions using CHARMM36-QMcharges-Literature parameters, where QM charges are taken from literature.[1] All systems contain 200 lipids, have Cl^- counter ions, and were simulated at 298 K.

**TPP concentration**[a] **[mM]**	**Length [ns]**	**Equil.**[b] **[ns]**	**TPP**[c]**/ Cl^-** [c]	**Waters**[c]	**Repeat distance**[d] **[nm]**	**APL**[d] **[nm^2]**	**Ref.**[e]
138	1 472	400	50	20 000	13.6	0.64	31
277	1 410	200	100	20 000	13.9	0.64	32
416	1 490	600	150	20 000	14.2	0.65	33

a) Calculated as TPP/waters * 55.5 * 1000.
b) Beginning part of the simulation left out as equilibration.
c) Number of molecules in the system.
d) Analysed from the equilibrated part.
e) The number of reference where simulation files are available.

Table S11: Simulated POPC bilayer system with tetraphenylphosphonium ions using CHARMM36-QMcharges-Literature-ECC parameters, where QM charges are taken from literature.[1] All systems contain 200 lipids, have Cl^- counter ions, and were simulated at 298 K.

**TPP concentration**[a] **[mM]**	**Length [ns]**	**Equil.**[b] **[ns]**	**TPP**[c]**/ Cl^-** [c]	**Waters**[c]	**Repeat distance**[d] **[nm]**	**APL**[d] **[nm^2]**	**Ref.**[e]
138	1442	400	50	20 000	12.4	0.7	34
277	1372	400	100	20 000	12.6	0.71	35
416	1158	500	150	20 000	12.8	0.72	36

a) Calculated as TPP/waters * 55.5 * 1000.
b) Beginning part of the simulation left out as equilibration.
c) Number of molecules in the system.
d) Analysed from the equilibrated part.
e) The number of reference where simulation files are available.

SMS cyclic peptide

Table S12: Simulated POPC bilayer system with SMS cyclic peptide using CHARMM36-ProteinFF parameters. All systems contain 200 lipids, have Cl^- counter ions, and were simulated at 298 K.

**SMS concentration**[a] **[mM]**	**Length [ns]**	**Equil.**[b] **[ns]**	**SMS**[c]**/ Cl^-** [d]	**Waters**[c]	**Repeat distance**[e] **[nm]**	**APL**[e] **[nm^2]**	**Ref.**[g]
12	284	100	2	9 202	8.1	0.64	—
24	291	140	4	9 016	8.0	0.65	—
32	277	50	6	10 192	8.5	0.65	—
39	2 166	2 166	18	25 234	—	—	37
41[f]	1 365	0	15	20 000	12.2	0.70	38
45	270	150	8	9 698	8.1	0.67	—
58	548	100	10	9 535	8.0	0.67	—
74	360	190	12	8 991	7.7	0.69	—
78	754	350	14	9 948	8.1	0.70	—
93	281	281	16	9 463	—	—	—

a) Calculated as SMS/waters * 55.5 * 1000.

b) Beginning part of the simulation left out as equilibration.

c) Number of molecules in the system.

d) Number of Cl^- is double of SMS^{2+}

e) Analysed from the equilibrated part.

f) All SMS molecules were bound at the beginning.

g) The number of reference where simulation files are available.

Dibucaine

Table S13: Simulated POPC bilayer system with dibucaine using CHARMM36-SwissParam parameters. All systems contain 200 lipids, have Cl^- counter ions, and were simulated at 298 K.

**Dibucaine concentration**[a] [mM]	**Length** [ns]	**Equil.**[b] [ns]	**DIB**[c]/ Cl^- [c]	**Waters**[c]	**Repeat distance**[d] [nm]	**APL**[d] [nm^2]	**Ref.**[e]
12	305	50	2	8 873	8.0	0.64	—
15	241	0	48	177 600	76.6	0.72	39
24	295	50	4	9 022	8.0	0.64	—
52	288	100	8	8 455	7.7	0.65	—
78	251	50	12	8 506	7.7	0.66	—
98	284	50	16	9 059	7.9	0.66	—
118	244	50	20	9 372	8.0	0.67	—
123	114	50	100	45 000	25.7	0.64	40
148	581	50	24	8 996	7.9	0.67	—
177	586	100	28	8 731	7.6	0.68	—
193	285	150	32	9 160	7.8	0.69	—
213	292	50	36	9 350	7.8	0.70	—
237	287	50	40	9 347	7.7	0.70	—
249	211	50	44	9 787	7.9	0.71	—
284	138	20	48	9 356	7.7	0.71	—

a) Calculated as DIB/waters * 55.5 * 1000.

b) Beginning part of the simulation left out as equilibration.

c) Number of molecules in the system.

d) Analysed from the equilibrated part.

e) The number of reference where simulation files are available.

References

(1) Schamberger, J.; Clarke, R. J. Hydrophobic ion hydration and the magnitude of the dipole potential. *Biophys. J.* **2002**, *82*, 3081–3088.

(2) Catte, A.; Girych, M.; Javanainen, M.; Loison, C.; Melcr, J.; Miettinen, M. S.; Monticelli, L.; Maatta, J.; Oganesyan, V. S.; Ollila, O. H. S.; Tynkkynen, J.; Vilov, S. Molecular electrometer and binding of cations to phospholipid bilayers. *Phys. Chem. Chem. Phys.* **2016**, *18*, 32560–32569.

(3) Javanainen, M.; Melcrová, A.; Magarkar, A.; Jurkiewicz, P.; Hof, M.; Jungwirth, P.; Martinez-Seara, H. Two cations, two mechanisms: interactions of sodium and calcium with zwitterionic lipid membranes. *ChemComm* **2017**, *53*, 5380–5383.

(4) Dias, C. L.; Jalali, S.; Yang, Y.; Cruz, L. Role of Cholesterol on Binding of Amyloid Fibrils to Lipid Bilayers. *J. Phys. Chem. B* . **2020**, *124*, 3036–3042.

(5) Clarke, R. J. The dipole potential of phospholipid membranes and methods for its detection. *Adv. Colloid Interface Sci.* **2001**, *89-90*, 263–281.

(6) Nencini, R. Etidocaine binding to POPC membrane, CHARMM36-SwissParam, 69 mM, 298 K . 2022; `https://doi.org/10.5281/zenodo.7010061`, Online; accessed 19 August 2022.

(7) Nencini, R. Etidocaine binding to POPC membrane, CHARMM36-SwissParam, 138 mM, 298 K . 2022; `https://doi.org/10.5281/zenodo.7010083`, Online; accessed 19 August 2022.

(8) Nencini, R. Etidocaine binding to POPC membrane, CHARMM36-SwissParam, 138 mM, 298 K, started from overbound . 2022; `https://doi.org/10.5281/zenodo.7010087`, Online; accessed 19 August 2022.

(9) Nencini, R. Etidocaine binding to POPC membrane, CHARMM36-SwissParam, 138 mM, 298 K, 177 600 waters . 2022; https://doi.org/10.5281/zenodo.7010110, Online; accessed 19 August 2022.

(10) Nencini, R. Etidocaine binding to POPC membrane, CHARMM36-SwissParam, 280 mM, 298 K . 2022; https://doi.org/10.5281/zenodo.7010119, Online; accessed 19 August 2022.

(11) Nencini, R. Etidocaine binding to POPC membrane, CHARMM36-SwissParam, 280 mM, 298 K, started from overbound . 2022; https://doi.org/10.5281/zenodo.7010136, Online; accessed 19 August 2022.

(12) Nencini, R. Etidocaine binding to POPC membrane, CHARMM36-SwissParam, 418 mM, 298 K . 2022; https://doi.org/10.5281/zenodo.7010152, Online; accessed 19 August 2022.

(13) Nencini, R. Etidocaine binding to POPC membrane, CHARMM36-SwissParam, 418 mM, 298 K, started from overbound . 2022; https://doi.org/10.5281/zenodo.7010154, Online; accessed 19 August 2022.

(14) Nencini, R. Etidocaine binding to POPC membrane, CHARMM36-ParamChem, 34 mM, 298 K . 2022; https://doi.org/10.5281/zenodo.6988344, Online; accessed 19 August 2022.

(15) Nencini, R. Etidocaine binding to POPC membrane, CHARMM36-ParamChem, 69 mM, 298 K . 2022; https://doi.org/10.5281/zenodo.7006630, Online; accessed 19 August 2022.

(16) Nencini, R. Etidocaine binding to POPC membrane, CHARMM36-ParamChem, 138 mM, 298 K . 2022; https://doi.org/10.5281/zenodo.6988407, Online; accessed 19 August 2022.

(17) Nencini, R. Etidocaine binding to POPC membrane, CHARMM36-ParamChem, 200 mM, 298 K . 2022; https://doi.org/10.5281/zenodo.7006630, Online; accessed 19 August 2022.

(18) Nencini, R. Etidocaine binding to POPC membrane, CHARMM36-ParamChem-ECC, 138 mM, 298 K . 2022; https://doi.org/10.5281/zenodo.7006441, Online; accessed 19 August 2022.

(19) Nencini, R. TPP binding to POPC membrane, CHARMM36-ProteinFF, 138 mM, 298 K . 2022; https://doi.org/10.5281/zenodo.6998195, Online; accessed 18 August 2022.

(20) Nencini, R. TPP binding to POPC membrane, CHARMM36-ProteinFF, 277 mM, 298 K . 2022; https://doi.org/10.5281/zenodo.6998201, Online; accessed 18 August 2022.

(21) Nencini, R. TPP binding to POPC membrane, CHARMM36-ProteinFF, 416 mM, 298 K . 2022; https://doi.org/10.5281/zenodo.6998205, Online; accessed 18 August 2022.

(22) Nencini, R. TPP binding to POPC membrane, CHARMM36-ProteinFF-ECC, 138 mM, 298 K . 2022; https://doi.org/10.5281/zenodo.6998211, Online; accessed 18 August 2022.

(23) Nencini, R. TPP binding to POPC membrane, CHARMM36-ProteinFF-ECC, 277 mM, 298 K . 2022; https://doi.org/10.5281/zenodo.6998215, Online; accessed 18 August 2022.

(24) Nencini, R. TPP binding to POPC membrane, CHARMM36-ProteinFF-ECC, 416 mM, 298 K . 2022; https://doi.org/10.5281/zenodo.7000346, Online; accessed 18 August 2022.

(25) Nencini, R. TPP binding to POPC membrane, CHARMM36-QMcharges, 138 mM, 298 K . 2022; https://doi.org/10.5281/zenodo.7000370, Online; accessed 18 August 2022.

(26) Nencini, R. TPP binding to POPC membrane, CHARMM36-QMcharges, 277 mM, 298 K . 2022; https://doi.org/10.5281/zenodo.7000382, Online; accessed 18 August 2022.

(27) Nencini, R. TPP binding to POPC membrane, CHARMM36-QMcharges, 416 mM, 298 K . 2022; https://doi.org/10.5281/zenodo.7000394, Online; accessed 18 August 2022.

(28) Nencini, R. TPP binding to POPC membrane, CHARMM36-QMcharges-ECC, 138 mM, 298 K . 2022; https://doi.org/10.5281/zenodo.7000418, Online; accessed 18 August 2022.

(29) Nencini, R. TPP binding to POPC membrane, CHARMM36-QMcharges-ECC, 277 mM, 298 K . 2022; https://doi.org/10.5281/zenodo.7000434, Online; accessed 18 August 2022.

(30) Nencini, R. TPP binding to POPC membrane, CHARMM36-QMcharges-ECC, 416 mM, 298 K . 2022; https://doi.org/10.5281/zenodo.7000446, Online; accessed 18 August 2022.

(31) Nencini, R. TPP binding to POPC membrane, CHARMM36-QMcharges-Literature, 138 mM, 298 K . 2022; https://doi.org/10.5281/zenodo.7000458, Online; accessed 18 August 2022.

(32) Nencini, R. TPP binding to POPC membrane, CHARMM36-QMcharges-Literature, 277 mM, 298 K . 2022; https://doi.org/10.5281/zenodo.7000468, Online; accessed 18 August 2022.

(33) Nencini, R. TPP binding to POPC membrane, CHARMM36-QMcharges-Literature, 416 mM, 298 K . 2022; https://doi.org/10.5281/zenodo.7000483, Online; accessed 18 August 2022.

(34) Nencini, R. TPP binding to POPC membrane, CHARMM36-QMcharges-Literature-ECC, 138 mM, 298 K . 2022; https://doi.org/10.5281/zenodo.7000500, Online; accessed 18 August 2022.

(35) Nencini, R. TPP binding to POPC membrane, CHARMM36-QMcharges-Literature-ECC, 277 mM, 298 K . 2022; https://doi.org/10.5281/zenodo.7000519, Online; accessed 18 August 2022.

(36) Nencini, R. TPP binding to POPC membrane, CHARMM36-QMcharges-Literature-ECC, 416 mM, 298 K . 2022; https://doi.org/10.5281/zenodo.7000541, Online; accessed 18 August 2022.

(37) Nencini, R. SMS binding to POPC membrane, CHARMM36-ProteinFF, 39 mM, 298 K . 2022; https://doi.org/10.5281/zenodo.7014209, Online; accessed 22 August 2022.

(38) Nencini, R. SMS binding to POPC membrane, CHARMM36-ProteinFF, 41 mM, 298 K, started from overbound . 2022; https://doi.org/10.5281/zenodo.7014213, Online; accessed 22 August 2022.

(39) Nencini, R. Dibucaine binding to POPC membrane, CHARMM36-SwissParam, 15 mM, 298 K, started from overbound . 2022; https://doi.org/10.5281/zenodo.7014252, Online; accessed 22 August 2022.

(40) Nencini, R. Dibucaine binding to POPC membrane, CHARMM36-SwissParam, 123 mM, 298 K . 2022; https://doi.org/10.5281/zenodo.7014254, Online; accessed 22 August 2022.
